# The activity and expression of adenylosuccinate lyase were reduced during modern human evolution, affecting brain and behavior

**DOI:** 10.1073/pnas.2508540122

**Published:** 2025-08-04

**Authors:** Xiang-Chun Ju, Shin-Yu Lee, Richard Ågren, Luiz Carlos Machado, Jiawei Xing, Chika Azama, Michael C. Roy, Toshihiro Endo, Wieland Huttner, Adam Siepel, Izumi Fukunaga, Hugo Zeberg, Svante Pääbo

**Affiliations:** ^a^Okinawa Institute of Science and Technology Graduate University, Okinawa 904-0495, Japan; ^b^Department of Physiology and Pharmacology, Karolinska Institutet, Stockholm 17177, Sweden; ^c^Simons Center for Quantitative Biology, Cold Spring Harbor, NY 11724; ^d^Phenovance, Kashiwa 277-0882, Japan; ^e^Max Planck Institute of Molecular Cell Biology and Genetics, Dresden 01307, Germany; ^f^Max Planck Institute for Evolutionary Anthropology, Leipzig 04103, Germany

**Keywords:** purine biosynthesis, human evolution, adenylosuccinate lyase

## Abstract

Adenylosuccinate lyase (ADSL), an enzyme that is crucial for purine biosynthesis, carries an amino acid substitution that is present in almost all humans today but absent in Neandertals and Denisovans. This substitution reduces the stability of the enzyme, but what functional consequences it has are unknown. Here, we show that when introduced into mice, this substitution causes substrates of the enzyme to accumulate in amounts that correlate negatively with ADSL expression levels. In the brain, where the expression of the enzyme is low, the substitution results in particularly high substrate levels. When the behavior of the mice is analyzed, female mice expressing the modern human-like version of ADSL access water more efficiently for drinking than their wild-type littermates. In addition to the amino acid substitution, a haplotype in the ADSL gene occurs at a carrier frequency of >97% in present-day humans and exhibits evidence of positive selection. It is associated with less ADSL expression as well as with increased concentrations of succinyladenosine, one of the substrates of the enzyme, in cerebrospinal fluid. Thus, two genetic changes have reduced ADSL activity in human tissues since modern and archaic humans separated, affecting purine biosynthesis, particularly in the brain.

## Significance

An amino acid substitution and noncoding changes have reduced adenylosuccinate lyase activity in modern humans after the divergence from Neandertals and Denisovans. This resulted in increased concentrations of purine substrates of the enzyme, particularly in the brain. When introduced into mice, the amino acid substitution causes females to access water more readily than their wild-type littermates.

Modern humans diverged from their closest evolutionary relatives, Neandertals and Denisovans, approximately 600,000 y ago (1). Since that time, a comparatively small number of genetic changes appeared on the modern human lineage and spread to almost all present-day humans (1–3). Some of these changes may be involved in phenotypic traits that set modern humans apart from earlier forms of humans. Among such traits, those that affect the brain and behavior may be most consequential as modern humans differ from now extinct human forms in that they have developed complex technology and culture that change rapidly (4, 5).

To date, relatively few genetic changes that are present in most modern humans and not in Neandertals and Denisovans have been characterized functionally (3). Three amino acid substitutions in the proteins KNL1 and KIF18A, and one amino acid substitution in the enzyme TKTL1, have been shown to increase the length of metaphase during stem cell division during brain development (6) and the number of cortical neurons generated (7), respectively. The RNA-binding protein NOVA1 carries an amino acid substitution that has been reported to affect morphology, synaptic protein expression, and electrophysiology in human brain organoids (8, but see refs. 9 and 10) as well as RNA splicing and vocalization when introduced into the mice (11). Another substitution in the enzyme glutathione reductase decreases the production of oxidative radicals under certain conditions (12), while one amino acid substitution in the aryl hydrocarbon receptor (AHR) reduces its ability to induce the expression of its target genes (13). However, the extent to which most of these changes affect behavior is not known.

**Fig. 1.**
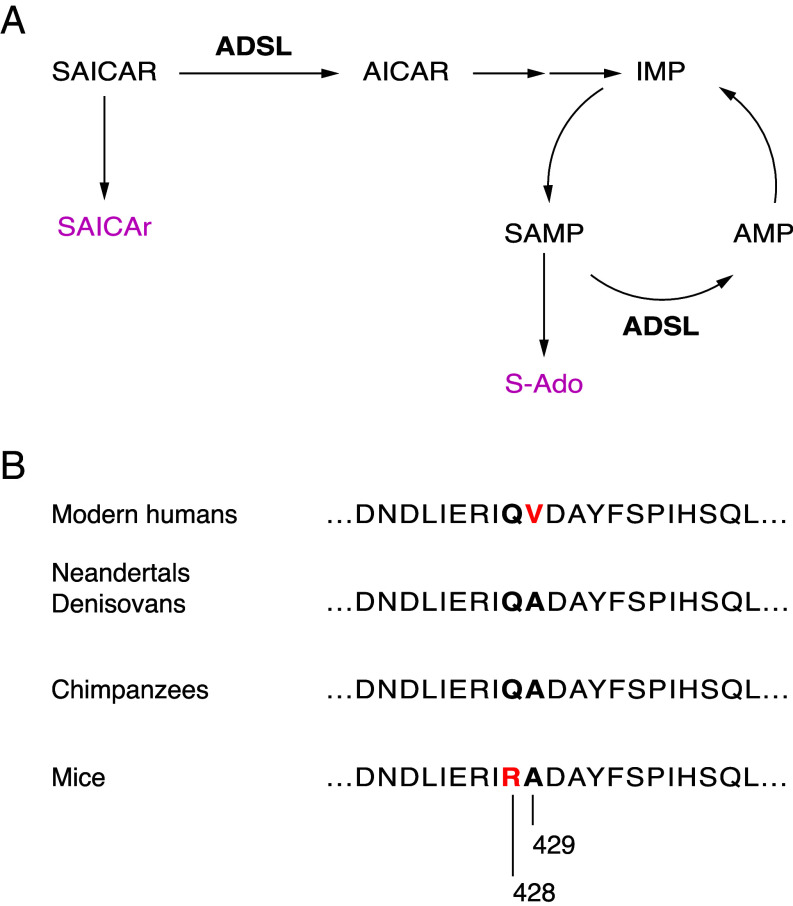
ADSL enzymatic activities and protein sequence. (*A*) The ADSL enzyme catalyzes two reactions in purine biosynthesis. The levels of the dephosphorylated forms of the ADSL substrates (red), SAICAr and S-Ado, are elevated when ADSL activity is decreased. SAICAR: succinylaminoimidazole carboxamide ribotide; SAICAr: succinylaminoimidazole carboxamide riboside; AICAR: aminoimidazole carbozamide ribotide; IMP: inosine monophosphate; SAMP: adenylosuccinate; S-Ado: succinyladenosine; AMP: adenine monophosphate. (*B*) Partial amino acid sequences surrounding position 429 in the ADSL protein.

Previous work (14, 15) has shown that the enzyme adenylosuccinate lyase (ADSL), which catalyzes two steps in purine biosynthesis (Fig. 1*A*), carries an amino acid substitution specific to modern humans (A429V) and reduces the stability of the enzyme in vitro. The A429V substitution has risen to high frequencies in modern humans. For example, the ancestral variant is absent among 821,979 exomes in the Regeneron Genetics Center database (https://rgc-research.regeneron.com/me/home) and is seen in a single case among 1.6 million genomes currently available in the gnomAD database (16). The *ADSL* gene is located in a genomic region that has undergone positive selection in modern humans after their divergence from Neandertals and Denisovans (17) and may therefore have conferred some advantage on modern humans early during their history.

When introduced into the mouse, the A429V substitution reduces ADSL enzymatic activity and the concentration of purines downstream of the reactions catalyzed by ADSL. Conversely, when the A429V substitution is reverted to its ancestral, Neandertal-like state in human cells, it increases the concentrations of downstream purines in the cells (14). Since genetic disorders causing ADSL deficiency in humans manifest with symptoms affecting the brain, for example, aggressive behavior, autism, intellectual disability, and speech and language impairment, (rarediseases.info.nih.gov), we decided to investigate the metabolic and behavioral effects of the A429V substitution more closely in the mouse as well as the evolution of the *ADSL* gene in humans.

## Results

### Metabolic Effects in the Mouse.

**Fig. 2.**
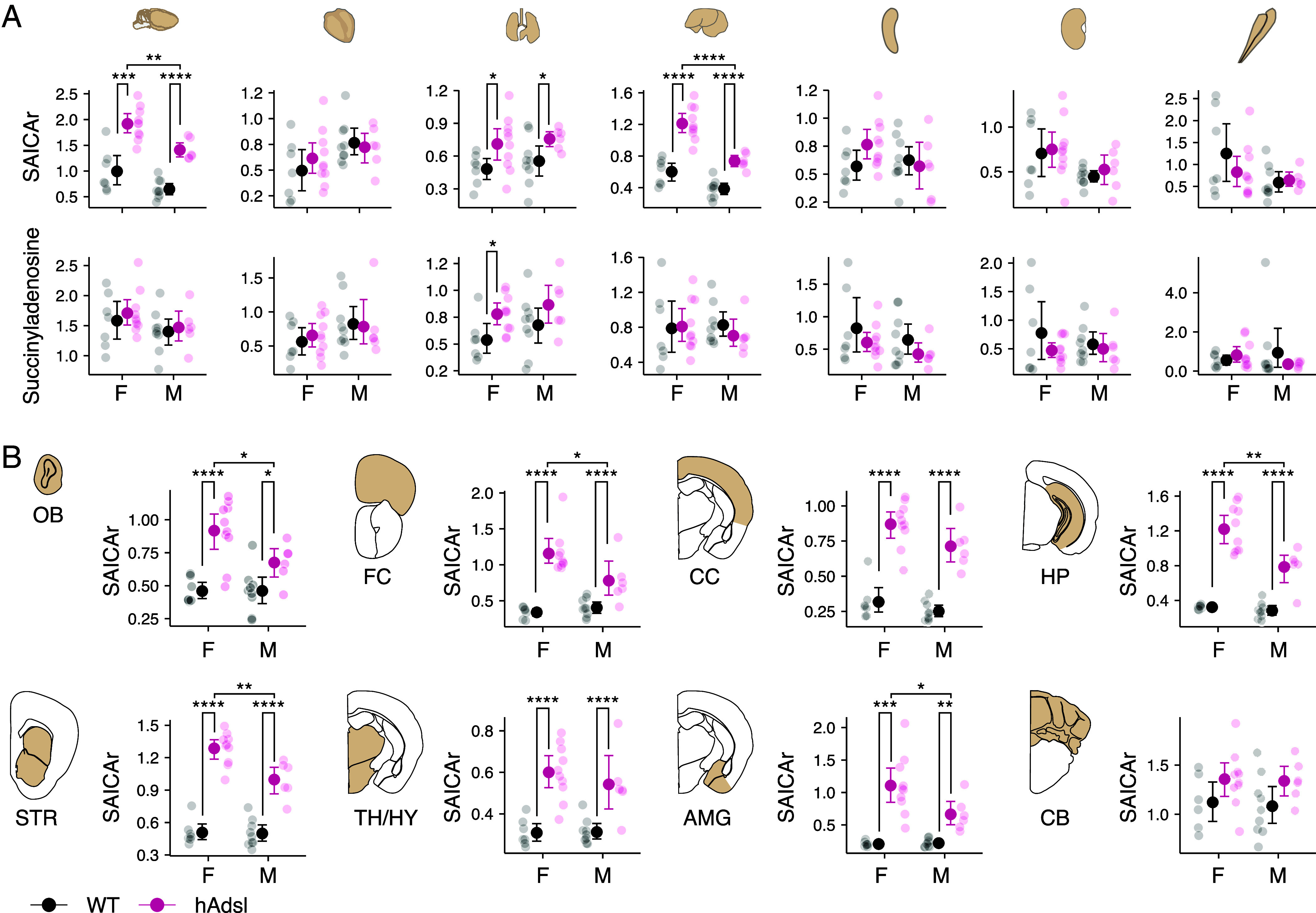
SAICAr and S-Ado concentrations in mice humanized for ADSL and their wild-type littermates. (*A*) Upper-quartile normalized SAICAr and S-Ado concentrations (y-axis) in various tissues of humanized Adsl mice (hAdsl, red) and their wild-type (WT, black) littermates (F: females. M: males). Tissues (*Left* to *Right*): forebrain, heart, lung, liver, spleen, kidney, and skeletal muscle. (*B*) Upper-quartile normalized SAICAr concentrations (y-axis) in 8 brain regions of hAdsl (red) mice and their WT (black) littermates. OB: olfactory bulb, FC: Frontal cortex, CC: cerebral cortex, HP: hippocampus, STR: striatum, TH/HY, thalamus and hypothalamus, AMG: amygdala, CB: cerebellum. For each group of mice, mean concentrations ± 95% CI as well as individual values are given. Student’s *t* test: *P < 0.05; **P < 0.01; ***P < 0.001; ****P < 0.0001.

To gauge the metabolic impact of the A429V amino acid replacement, we compared mice carrying the A429V substitution as well as an adjacent R428Q substitution that reverts a rodent-specific amino acid change to the general mammalian state with their wild-type littermates (Fig. 1*B*). In contrast to the change at position 429, the change at position 428 does not affect the protein stability in the mouse or the human ADSL backgrounds (14). The metabolic effects of these substitutions have so far been analyzed only regarding the tissue concentrations of purines several metabolic steps downstream of ADSL. To more directly gauge metabolic effects of the substitutions, we analyzed the two dephosphorylated forms of the substrates of ADSL, succinylaminoimidazole carboxamide riboside (SAICAr) and succinyladenosine (S-Ado), in seven organs (Fig. 2*A*).

The concentrations of SAICAr, the substrate of the first step in purine biosynthesis, which is catalyzed by ADSL, were higher in humanized mice than in their wild-type littermates in three of the organs analyzed. The increase is particularly pronounced in the brain and the liver where it is about twofold (Fig. 2*A* and *SI Appendix,* Tables S1 and S2). The brain stands out in that the absolute concentrations of SAICAr as well as S-Ado, the substrate of the other reaction catalyzed by ADSL, is higher than in other tissues, particularly in female humanized mice.

To investigate whether the increase in SAICAr concentrations induced by the amino acid substitutions is specific to certain parts of the brain, we analyzed 8 different regions of the cerebrum as well as the cerebellum. In all regions of the cerebrum, SAICAr concentrations were increased 1.8- to 5.4-fold in the female mice that carry the “humanized” *Adsl* and 1.5- to 3.6-fold in the “humanized” males (Fig. 2*B* and *SI Appendix,* Table S3). In the cerebellum, the corresponding increases were more modest, 1.2- to 1.3-fold, and nominally significant only for the cerebellar cortex of the females (*SI Appendix,* Table S3).

To elucidate why the metabolic effects of the amino acid substitutions were more pronounced in certain organs than in others, we considered *Adsl* messenger RNA (mRNA) expression in mouse tissues (*SI Appendix,* Fig. S1 *A* and *B*). In organs such as the brain and liver where the humanized *Adsl* results in large SAICAr increases, the expression of *Adsl* mRNA was low, whereas in organs where small or no increases were seen, such as skeletal muscle or heart, the expression of *Adsl* was high. Thus, the increase in SAICAr concentrations in the mice expressing the humanized form of ADSL correlates negatively with the expression of *Adsl* mRNA (R = −0.78; *SI Appendix,* Fig. S2*A*). Within the brain, *Adsl* mRNA expression is similarly negatively correlated with the SAICAr increase (R = −0.94; *SI Appendix,* Fig. S2*B*) and negatively correlated with the S-Ado increase (R = −0.84; *SI Appendix,* Fig. S2*C*). This suggests that the reduction in ADSL activity caused by the modern human-specific amino acid substitution has greater consequences in tissues where the expression of the enzyme is low.

Since the metabolic effects in the mice humanized for ADSL were more pronounced in female than in male mice, we explored whether the *Adsl* mRNA or protein expression may differ between the sexes and between genotypes. There was no difference in *Adsl* mRNA expression in either brain or muscle, neither between the sexes nor between the genotypes (*SI Appendix,* Fig. S3 *A* and *B*). In the brain, ADSL protein expression was too low to be detected. However, in the muscle, where the enzyme could be detected in immunoblots, female humanized mice contained ~40% less protein than their male littermates (*P* < 0.01) (*SI Appendix,* Fig. S3*C*), compatible with that lower enzyme levels in the humanized female mice make them more sensitive than the males to a reduction in enzymatic activity.

### Behavioral Effects in the Mouse.

Since the metabolic effects of the *Adsl* mutations were particularly pronounced in the brain, we explored the behavior of mice homozygous for the modern human-like substitutions and their wild-type littermates in the IntelliCage system (18, 19). This system is often used to automatically record mouse behavior in their home cages, thus reducing the extent to which human handling and experimental setups may influence mouse behavior. Five to eight mice of each genotype and the same sex were housed together in cages where over a period of 1 wk they learned to drink water by poking their noses in devices in the four corners of the cage. Mice of each genotype learned this equally fast and visited the rewarded corners equally frequently (*SI Appendix,* Fig. S4).

**Fig. 3.**
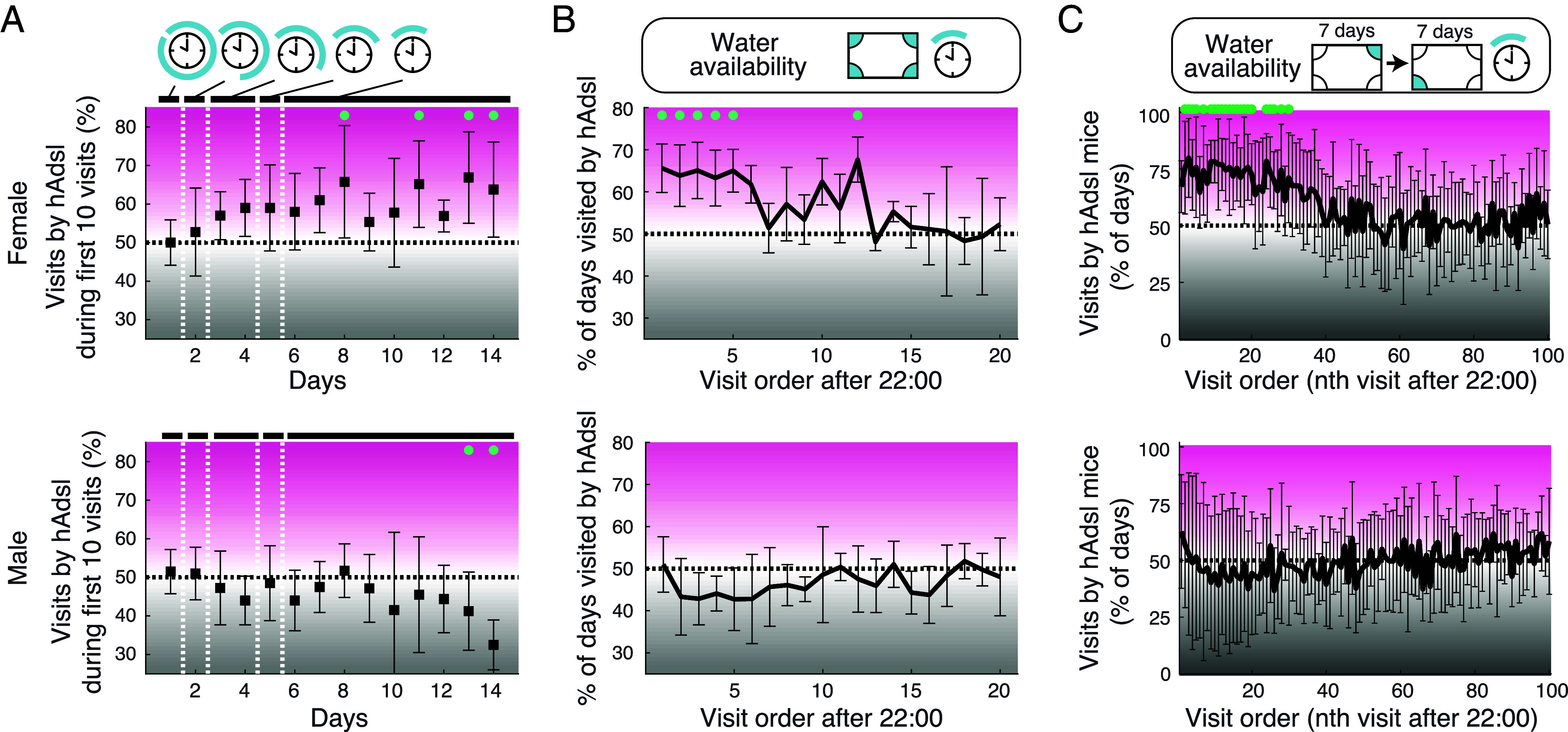
Female mice humanized for ADSL access water quicker after water restriction. (*A*) The proportion of visits by hAdsl (humanized) mice among the first 10 visits at the four corners on each day as the water was progressively restricted (12, 8, 6, 4, 3 h a day). *Upper* and *Lower* panels correspond to female and male cages (5 each). Mean ± SD across cages are shown. Green dots indicate significant differences from shuffled datasets at α = 0.05 level (two-tailed test with Bonferroni correction). A dotted horizontal line at 50% indicates equal access by hAdsl and WT (wild-type) mice. (*B*) The proportion of days on which hAdsl mice visited the four corners first when water became available (at 22:00). The total number of days was 9 d for each cage. (*C*) The percentage of days upon which hAdsl mice visited the indicated corners after water became available at 22:00. The total number of days analyzed was 14 d for each cage.

Over 12 d, the access to water was progressively restricted from 12 h to 3 h in the night when the mice are active. A sound signal as well as lights at the four corners announced the start of the availability of the water. Initially, humanized and wild-type individuals were equally likely to visit the corners and drink when water became available. However, from day three onward, the female humanized mice made more visits to the corners than their wild-type littermates when water became available (Fig. 3*A*). For example, from 6 d onward, when water was available for 3 h, there were 1.8 times more visits by humanized females than by wild-type females when considering the first five visits (*P* = 8.2 × 10^−4^, two-tailed test against shuffled distribution). A difference between the genotypes remained for the first ~12 visits (Fig. 3*B*). No such difference between the genotypes was observed for the male mice.

The access to water was then further restricted by making it available only at one, rather than four, corners. There were again more early visits by humanized than by wild-type females, and this difference now persisted for the first ~30 visits. For example, among the first 20 visits, humanized females visited the corner where water was available 2.8 times more often than their wild-type littermates (*P* = 0.019, two-tailed test against shuffled distribution) (Fig. 3*C*). This difference was present consistently from the first day of this reduced regime. It was seen also after the corner where water was available was changed to the opposite side of the cage. Again, there was no difference between the genotypes among the male mice (Fig. 3*C* and *SI Appendix,* Fig. S5*A*). Mice of both genotypes and both sexes did not differ in how often they visited the three unrewarded corners (*SI Appendix,* Fig. S5*B*), indicating that all mice moved around the cages to the same extent. In conclusion, female mice that carry the human-like amino acid residues at positions 428 and 429 compete more efficiently for water when it is a desirable resource than their wild-type littermates.

To investigate whether the humanized mice show dominant behavior when not competing for a scarce resource such as water, we housed groups of two humanized mice together with two of their wild-type, weight-matched littermates. After 14 d, an experimenter, who was blinded to the genotypes of the mice, tested all six pairs of mice by placing them in the opposite ends of a narrow acrylic tube. In this test, the mouse that goes through the entire length of the tube, pushing the other out, is defined as a “winner” (20). There were no differences in how often the humanized and the wild-type mice of either sex were “winners” (*SI Appendix,* Fig. S6). Thus, in this experimental paradigm, the humanized mice are not intrinsically dominant.

Since *Adsl* is most highly expressed in the skeletal muscle (*SI Appendix,* Fig. S1*A*) and since muscle performance might influence how well the mice compete for water, we tested whether there is a difference between the humanized and wild-type mice, using three tests of muscle strength and two tests of running ability (*SI Appendix,* Fig. S7). Male humanized mice had a slightly greater grip strength than their wild-type littermates (13%, *P* = 0.001), but there was no difference in two other tests of muscle strength, nor in terms of speed or endurance when running. Thus, there is no indication that the humanized females would be faster or stronger than their wild-type littermates.

### ADSL Expression and Activity in Humans.

**Fig. 4.**
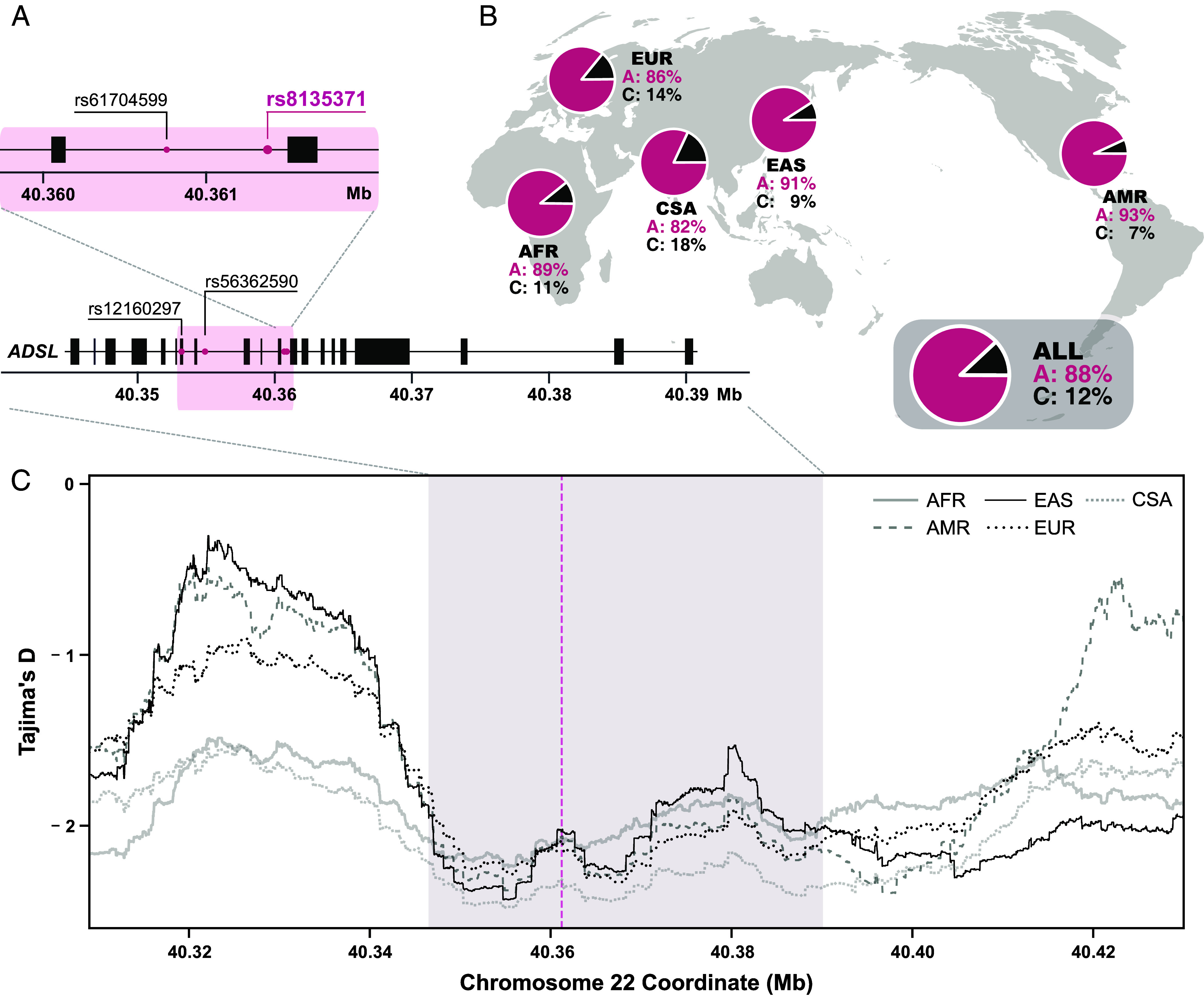
A haplotype associated with lower ADSL expression in present-day humans. (*A*) ADSL exon map and the four variants forming a 7.8-kb haplotype (shadowed area). (*B*) Frequencies of the rs8135371 alleles across five continental populations: AFR (African), AMR (Admixed American), CSA (Central/South Asian), EAS (East Asian), and EUR (European), with ALL indicating the combined dataset. (*C*) Tajima’s D (excess of rare alleles) in different populations in the region of the ADSL gene (shadowed area). The dotted line indicates the rs8135371 variant position.

We next explored whether any other genetic variants in the *ADSL* gene of humans may affect its expression, perhaps compensating for the reduction in activity caused by the A429V amino acid substitution. We find that eleven other nucleotide changes in the *ADSL* gene have arisen in modern humans and reached frequencies higher than 80%. Four of these create a haplotype of 7.8 kilobases (Fig. 4*A*) and are likely to have regulatory consequences (*SI Appendix,* Fig. S8). One of the variants in this haplotype (rs8135371) is a single-nucleotide polymorphism where a novel A nucleotide appeared in modern humans (chr22:40361224-A/C; GRCh38.p14, rs8135371; Variant Effect Prediction; Ensembl release 112). The A variant is associated with lower *ADSL* mRNA expression in many tissues (*SI Appendix,* Fig. S9). This is most pronounced in the cerebrum (*SI Appendix*, Fig. S9, *P* < 1e−6). The effect is also large in testis but smaller in other tissues. In agreement with this, proteomic datasets (21) show that carriers of the A variant have ~7% lower levels of ADSL protein in the brain than carriers of the C variant (*P* < 1e−5). Furthermore, among 31,684 blood samples (22) the A variant is associated with lower *ADSL* mRNA expression (*P* = 8.6e−85). Thus, rather than compensating for the lower ADSL activity caused by the A429V amino acid substitution by increasing *ADSL* expression, the haplotype carrying the A variant at rs8135371 lowers *ADSL* expression, particularly in the brain.

The A variant occurs at an allele frequency of 82 to 93% in all major populations of the world (Fig. 4*B*), indicating that 97% or more of people today carry at least one copy of the variants that decrease *ADSL* expression. A measure of the excess of rare alleles (23) indicates that they are more common in a region of approximately 10,000 nucleotides encompassing the haplotype in which rs8135371 is located than in surrounding chromosomal regions. This signal, which is seen in all major populations of the world and reaches a numerical value below −2 (Fig. 4*C*), is compatible with that the haplotype has risen in frequency due to positive selection within the last 250,000 y (24).

To more directly investigate whether positive selection has affected the *ADSL* gene, we used ARGweaver-D (25) to reconstruct ancestral recombination graphs (ARG) for modern and archaic hominins in the genomic region surrounding *ADSL*, incorporating sequences for modern African, European, and East Asian individuals and three Neandertal and one Denisovan genome. We then sampled local genealogies for two haplotypes—one harboring the derived alleles for SNPs rs12160297, rs56362590, and rs61704599, and another the derived allele for SNP rs8135371—and tested them for evidence of positive selection using the likelihood-based CLUES2 method (26). This approach considers many possible trajectories of haplotype frequency over time, weighting them by their relative probabilities given the observed DNA sequences, and allowing for uncertainty in coalescence times, recombination events, and selection pressures.

Under these tests, both haplotypes showed strong evidence of positive selection, with an estimated selection coefficient of *s* = 0.0018 (*P* = 0.009 for the null hypothesis of no selection) for the first haplotype and an estimated selection coefficient of *s* = 0.0016 (*P* = 0.015) for the second haplotype. These estimates correspond to population-scaled selection coefficients of ~36 and ~32, respectively, many times greater than the conventional threshold of one for nearly neutral evolution (27). The estimated selection coefficients and –log *P*-values were substantially elevated relative to an empirical null distribution based on noncoding variants matched in allele frequency but likely to be free from positive selection (*SI Appendix,* Fig. S10). Inspection of the inferred genealogies revealed a burst of recent coalescence events and reduced times to most recent common ancestry at *ADSL* in comparison to more diffuse patterns of coalescence and more ancient ancestry at control loci, as would be expected from a rapid rise in allele frequency in early modern humans owing to positive selection (*SI Appendix,* Fig. S11).

In agreement with that the *ADSL* haplotypes have risen in frequency due to positive selection in the more distant past, the frequency of the derived haplotype has remained stable at ~90% in Europe over the past 10,000 y (*SI Appendix,* Fig. S12). This is compatible with the strength of selection estimated above which may be on the same level as random genetic drift over these time periods but also, for example, with that selection may have become irrelevant in human societies after the introduction of agriculture.

**Fig. 5.**
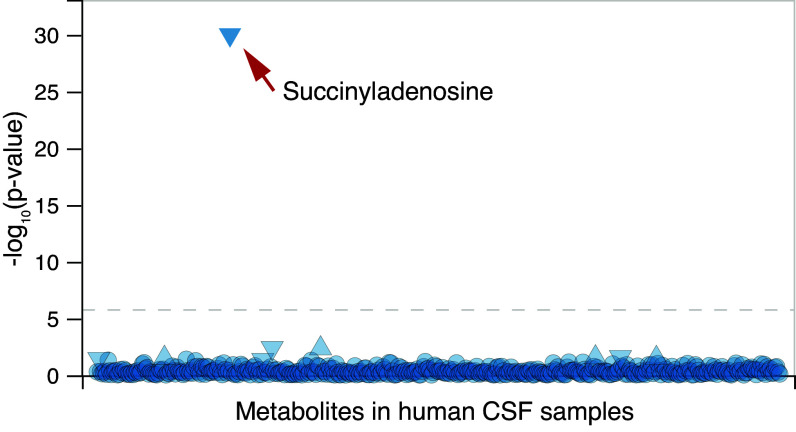
Associations of rs8135371 with 440 metabolites in 2,602 human cerebrospinal fluid samples. Dashed line: *P*-value = 1.9 × e^−6^. Data from the ONTIME web portal (https://ontime.wustl.edu/).

In individuals affected by ADSL deficiency, elevated levels of SAICAr and S-Ado in cerebrospinal fluid (CSF) reflect lower ADSL activity in the brain (28). We therefore investigated a recent dataset where 440 metabolites, including S-Ado (but not SAICAr), were analyzed in the CSF of 2,602 individuals for which genome-wide genetic data are available (29). The polymorphism that shows the most significant genome-wide association with CSF levels of S-Ado is rs8135371 (*P* = 8.5e−31) (Fig. 5). Carriers of the A variant have higher levels of S-Ado (beta = 0.052, SE = 0.0045), indicating that this allele is associated with lower ADSL activity in the brain.

Given that S-Ado as well as SAICAr levels have increased in the brains of modern humans and that S-Ado is structurally similar to adenosine, we tested whether the substrates of ADSL may exert effects on adenosine receptors by transcribing complementary DNAs (cDNAs) encoding the human adenosine A1, A2A, and A3 receptors as well as two G-protein-coupled potassium channels and injecting them into *Xenopus laevis* oocytes. After 6 d, all three receptors responded to adenosine, while no response was detected for SAICAR or S-Ado (*SI Appendix,* Fig. S13), indicating that neither substrate of ADSL exerts functions by directly binding to adenosine receptors.

### Phenotypic Associations in Humans.

Given that ADSL deficiency is characterized by both elevated levels of S-Ado and intellectual disability, we explored whether common genetic variants associated with S-Ado levels are correlated with cognitive abilities. To this end, we used the largest genetic meta-analysis of intelligence to date (N = 269,867; 30) and the genetic association study of S-Ado levels in the cerebrospinal fluid of 2,602 individuals (29). We employed the technique of genetic correlation, which tests whether genetic variants associated with one trait are also associated with a second trait. We found a significant negative correlation between S-Ado levels in CSF and intelligence (r_g_ = −0.13, *P* = 0.0051). To gauge whether other phenotypes may also be associated with CSF levels of S-Ado, we analyzed a recent dataset that summarized the phenotypic variation in the UK Biobank into 35 “distilled” factors (31), but found no significant genetic correlation (*SI Appendix,* Table S4).

## Discussion

Two different genetic changes have reduced ADSL activity in modern humans after they separated from an ancestor shared with Neandertals and Denisovans. The first change was the amino acid substitution at position 429, which reduces the stability of the protein (14) and is present in essentially everybody today. The second change was noncoding nucleotide substitutions, which reduce the expression of the gene, and occurs in a homo- or heterozygous form in 97% of present-day people (Fig. 4).

The *ADSL* gene is located in an extended genomic region where Neandertals and Denisovans fall outside present-day human variation, suggesting that a selective sweep affected the region in modern human ancestors not long after the divergence of archaic and modern ancestors (17). This could have been caused by the amino acid substitution. The haplotype that reduces *ADSL* expression presumably rose to high frequencies later and has been affected by positive selection (*SI Appendix,* Figs. S10 and S11). ADSL activity in human tissues has thus been lowered twice during recent human evolution by genetic changes, both of which may have been positively selected.

Because ADSL is expressed in all human tissues, these genetic changes may have effects in any organ. However, the metabolic effects seem to be particularly pronounced in the brain, notably the cerebrum, and the liver (Fig. 2). We suggest that this is due to that *ADSL* expression is lower in the brain and liver than in other organs (*SI Appendix,* Fig. S1), making these organs more sensitive to reductions of ADSL activity. In agreement with the brain being particularly sensitive to reductions in ADSL activity, mutations that lower ADSL activity to pathological levels result in symptoms such as psychomotor retardation, aggressive behaviors, and seizures (rarediseases.info.nih.gov) (32). In addition, when the modern human A429V substitution was introduced into mice, we observed no effects in skeletal muscle (*SI Appendix,* Fig. S7) where ADSL expression is higher than in other organs (*SI Appendix,* Fig. S1). Instead, we observed behavioral effects in that mice carrying the modern human amino acid substitution competed more efficiently for water when they were thirsty. Strikingly, these effects are seen in female mice and not in male mice (Fig. 3). Consistent with the supposition that lower ADSL expression is associated with a greater sensitivity to reductions in the enzyme activity, female humanized mice express less ADSL protein than male humanized mice (*SI Appendix,* Fig. S3*C*).

The mechanism by which the changes in ADSL activity cause behavioral effects is unknown. Since the A429V substitution lowers the concentrations of several purines in the brains of mice (14), it could involve purinergic signaling, particularly of adenosine. For example, adenosine triphosphate released by astrocytes can suppress synaptic transmission (33), and adenosine released by neurons can affect astrocyte metabolism, in turn affecting synaptic plasticity and memory (34). It may also involve the substrates of ADSL. For example, S-Ado occurs at micromolar concentrations in the CSF (35) and is increased in humans carrying the derived variant of rs8135371 (Fig. 5). However, neither SAICAR nor S-Ado act as adenosine receptor agonists at relevant concentrations (*SI Appendix,* Fig. S13), making it unlikely that these compounds act directly on adenosine receptors.

An interesting question is what effects lower ADSL activity may have on humans today and in prehistoric times when positive selection may have occurred. The effects in the mouse model, for example, the water competition task, cannot directly be translated to human phenotypes. However, the fact that S-Ado levels in the CSF are negatively associated with intelligence suggests that lowered ADSL activity may affect brain functions, although effects in other tissues cannot be excluded. Any selective effects would obviously be dependent on the genetic background as well as the environmental and cultural contexts in which the ADSL variants occur and may not be accurately reflected, for example, by intelligence tests as performed today. Future work should therefore address whether ADSL activity and/or S-Ado levels are associated with other traits in humans.

Another important aspect illustrated by this work is that several changes may be needed to more fully model human phenotypes in an experimental system such as the mouse. Often, several changes affecting a single gene product (e.g., ref. 36) or several gene products may be needed (e.g., ref. 6). In the case of ADSL, an amino acid change as well as noncoding changes affecting *ADSL* expression may be needed. In addition, those changes may exert their effects only in concert with other changes that affect, for example, purine metabolism or purinergic signaling. Thus, a promising approach to address modern human as well as archaic phenotypes in model organisms will be to combine several noncoding and coding changes that affect certain pathways, certain functional systems, or certain organelles in order to understand their combined effects.

## Materials and Methods

### Ethical Approval.

All mouse experiments have been approved by the OIST (Okinawa Institute of Science and Technology Graduate University) Animal Care and Use Committee (ACUP-2023-047-2 and ACUP-2022-017-3). Experiments on recombinant DNA were reviewed and approved by the OIST Biosafety Committee (RDE-2020-012-9). See *SI Appendix*, *Materials and Methods*.

### Generation of Adsl-Humanized Mice.

The generation of mice humanized for *Adsl* was conducted as described previously (14). Mice homozygous for the human-like ADSL and wild-type littermates were generated from heterozygous breeding pairs.

### Quantification of ADSL Enzymatic Substrates.

Tissues for metabolomics analyses were collected from 9- to 10-wk-old homozygous humanized and wild-type mouse littermates of both sexes and processed for metabolite extraction using a two-phase extraction protocol. Polar, water-soluble metabolites were separated by ultraperformance liquid chromatography and analyzed using a hybrid quadrupole-Orbitrap mass spectrometer. Upper-quartile normalized peak areas of the ADSL enzymatic substrates, SAICAr and S-Ado, were used for quantitation. See also *SI Appendix*, *Materials and Methods*.

### Mouse Behavioral Analysis.

Behavioral tests were conducted on homozygous humanized mice and wild-type littermates of both sexes. A fully automated IntelliCage system (TSE Systems GmbH, Bad Homburg, Germany) was used to assess “water competition” behaviors (18, 37). Mice were housed in the IntelliCage system with free access to water for 3 d prior to tests. Tube tests and muscle performance tests were performed on mice that were 18 to 25 and 8 to 13 wk old, respectively. The tube test was conducted according to a previously published protocol (38). The grip-strength test, weight test, and wire-hanging test were used to evaluate muscle strength (39–41). The sprint and endurance tests using a treadmill were adapted from established protocols (42, 43). See *SI Appendix*, *Materials and Methods*.

### mRNA Expression and Protein Analysis.

RNA extraction from brain, cDNA synthesis, and Western blot quantification of ADSL protein levels in muscle tissues were conducted using standard protocols as described in *SI Appendix*, *Materials and Methods*. The muscle tissue RNA extraction, library preparation, and sequencing were conducted at the OIST Sequencing Center, and the raw data are available via the Gene Expression Omnibus (GEO) repository (BioProject accession number: PRJNA1272784). The *ADSL* mRNA expression in human bulk tissues was retrieved from the GTEx portal “Expression” (dbGap accession number phs000424.v8.p2). *Adsl* mRNA expression in mouse tissues was retrieved from the GEO repository: GSE194203 (brain and nonbrain tissues) (44) and GSE178290 (male brains) (45). For immunoblotting, proteins were extracted from snap-frozen muscle samples. See *SI Appendix*, *Materials and Methods*.

### Selection Analysis.

The phased haplotypes from the 1000 Genomes Project and Human Genome Diversity Project were used to calculate Tajima’s D values (23). To investigate whether positive selection has affected the *ADSL* gene in humans, we reconstructed the ancestral recombination graphs (ARGs) using ARGweaver-D (25) and genome sequences from present-day and archaic humans. See *SI Appendix*, *Materials and Methods*.

### Adenosine Receptor–Ligand Binding Assay.

The bindings between three ligands, adenosine, S-Ado, and SAICAR, and four wild-type human adenosine receptors (A1, A2A, A2B, and A3) were analyzed in African clawed toad oocytes. See *SI Appendix*, *Materials and Methods*.

## Supplementary Material

Appendix 01 (PDF)

## Data Availability

The RNAseq data generated in this paper have been deposited in NCBI’s Sequence Read Archive (SRA) with the BioProject accession number PRJNA1272784 (46). All other data are included in the article and/or *SI Appendix*.
