# Supplementary material for: The activity and expression of adenylosuccinate lyase were reduced during modern human evolution, affecting brain and behavior
Source: Proc Natl Acad Sci U S A. 2025 Aug 4;122(32):e2508540122. doi: 10.1073/pnas.2508540122 (PMC12358879; doi:10.1073/pnas.2508540122)
Supplement: Supplementary file 1 — Appendix 01 (PDF) [file pnas.2508540122.sapp.pdf]

Supporting information for:

The activity and expression of adenylosuccinate lyase was reduced during modern human evolution, affecting brain and behavior

Xiang-Chun Ju*, Shin-Yu Lee, Richard Ågren, Luiz Carlos Machado, Jiawei Xing, Chika Azama, Michael C. Roy, Toshihiro Endo, Wieland Huttner, Adam Siepel, Izumi Fukunaga*, Hugo Zeberg*, Svante Pääbo*

*Corresponding authors:

Svante Pääbo
paabo@eva.mpg.de

Hugo Zeberg
hugo.zeberg@ki.se

Izumi Fukunaga
izumi.fukunaga@oist.jp

Xiang-Chun Ju
xiangchun.ju@oist.jp

This file includes:

Materials and Methods
Supplementary Figures 1 to 13 and legends
Supplementary Tables 1 to 4

Materials and Methods

Mice

All mouse experiments have been approved by the OIST (Okinawa Institute of Science and Technology Graduate University) Animal Care and Use Committee (ACUP-2023-047-2 and ACUP-2022-017-3). The AAALAC-certified committee comply with OIST regulations as stipulated in the Animal Experiment Protocol, Paragraph 1, Article 13 of the Animal Experiment Regulations at OIST and based on the Law for Humane Treatment and Management of Animals (Law No. 105, 1973). Experiments on recombinant DNA were reviewed and approved by the OIST Biosafety Committee (RDE-2020-012-9).

The generation of mice humanized for Adsl was conducted as described previously (1). Two amino acid changes (A429V, R428Q) were introduced into the humanized mice. All mice used in this study were offspring of heterozygous mouse parents on a C57BL/6N background. Up to five mice of the same sexes were cohoused in individually ventilated cages within SPF (specific pathogen free) areas on a 12:12-h (8:00/20:00) light:dark cycle.

Metabolic analysis

Tissues were collected from 9- to 10-week-old male and female homozygous Adsl-humanized (hAdsl: $N_{Female} = 10$, $N_{Male} = 6$) and homozygous wild-type (WT: $N_{Female} = 7$, $N_{Male} = 9$) littermates that had not been used in any study. Mice were deeply anesthetized using isoflurane before tissues were removed and snap-frozen in liquid nitrogen. Tissue samples were stored in a deep freezer (-80 °C) before dissection. Totally 16 anatomically different regions (OB, FRP, mPFC, MC, SSC, VC, AMG, NAc, dSTR, dHP, vHP, TH, HY, ENT, CBX, and CBN) and cerebral and cerebellar hemispheres were dissected on dry ice preventing thawing. Approximately 20 milligrams of six other tissues (heart, lung, liver, spleen, kidney, and hind limb skeletal muscle) were similarly dissected from the same mice.

During sample preparation and prior to analyses, lipids and polar water-soluble metabolites were separated through solvent extraction and fractionation. Briefly, 10 µL of caffeine solution (5 µg/mL,

Sigma-Aldrich, C6035) diluted in a 90% methanol-water-formic acid solution (MeOH: Sigma-Aldrich, 646377) was added into each 1.5-mL Eppendorf safe-lock tube with tissue samples or empty tubes without tissue samples as an internal control. The 90% methanol-water-formic acid solution comprised of 90% MeOH, 9.5% H_2O (Sigma-Aldrich, 34877), and 0.5% formic acid (FA: ThermoFisher Scientific, 28905). Tissues were then thoroughly homogenized in 100 µL of the above solution with an electric grinder (AS ONE, F65000-0000) and ultra-sonicated on an ice bath (2-4 °C) at the maximum power for 10 min. The homogenate was vortexed for 10 s and centrifuged at 14,000g at 4 °C for 5 min. The supernatant was transferred into a new 1.5-mL Eppendorf safe-lock tube, pre-cooled on dry ice. The tissue residue was extracted again using the above 90% MeOH (100 µL). The supernatant was transferred into the same first Eppendorf safe-lock tube. Water (200 µL) was added into the pooled extract tube and mixed well. Next, 400 µL of methyl tertiary-butyl ether (Sigma-Aldrich, 34875) was put into the above mixture (~400 µL) to induce phase separation. The mixture was vortexed for 10 s and shaked at room temperature (RT) at 1,500 rpm for 5 min. After centrifugation at 14,000×g at 4 °C for 5 min, the lower water (polar, ~320 µL) phase was collected and vacuum dried down using a centrifugal evaporator (Genevac EZ-2^{plus}). An aliquot (10 µL) of the remaining fractions were combined to form Quality Control (QC) pools. The QC pools were then injected after every set of 10 tissue samples when performing the liquid chromatography-mass spectrometry (LC-MS) analysis. Dried extracts were stored at -80 °C before being analyzed by LC-MS.

Immediately before the LC-MS analysis of polar metabolites, the dried polar phases were resuspended in a mixture of H_2O/MeOH/FA (89.5:10:0.5, v/v/v, 20 µL,). The mixture was ultra-sonicated in a water bath at RT for 3 min and then vortexed for 10 s. After centrifugation at 14,000×g at 4 °C for 10 min, the clean sample solution was collected (15 µL) in a LC-MS sample tube. The clean sample solution was injected (3 µL) into a Waters UPLC system equipped with a Waters Acquity UPLC HSS T3 column (150 mm × 1.0 mm i.d.; 1.8 µm). The oven temperature and flow rate were set at 40 °C and 50 µL/min, respectively. The mobile phases were water containing 0.1% FA (buffer A) and acetonitrile containing 0.1% FA (buffer B). A gradient run was set up as 0.0 - 2.1 min 1% B, 2.1 – 7.0 min from 1% to 35% B, 7.0 – 9.0 min keeping 35% B, 9.0 – 10.0 min from 35% to 98% B, 10.0 – 13.0

min washing 98% B, 13.0 – 13.1 min from 98% to 1% B, and 13.1 – 19.9 min re-equilibrating 1% B. A ThermoFisher Q-Extractive HF with a heated electrospray ionization (HESI) source (Thermo Scientific) was used to acquire mass spectra with a data-independent MS/MS spectra acquisition method and simultaneously in positive and negative modes. The HESI source conditions were set as follows: spray voltage, 3.0 kV for both modes; sheath gas flow rate, 30 arbitrary units; aux gas flow rate, 10 arbitrary units; sweep gas flow rate, 1 arbitrary unit; capillary temp, 300 °C; S-lens RF level, 30; Aux gas heater temperature, 150 °C. Full scan parameters: resolution, 60,000; Microscans, 5; Lock masses, 593.15761 $[M+H]^+$; AGC target, 3e6; Maximum IT, 50 ms; scan range 70–1,000 m/z; spectrum data type, profile. Data-independent MS^2 parameters: resolution, 30,000; AGC target, 5e5; maximum IT, 30 ms; loop count, 5; TopN, 5; isolation window, 4.0 m/z; fixed first mass, 50.0 m/z; (N)CE/stepped nce, 20, 30, 40 (collision energies are normalized to m/z 500, z = 1); spectrum data type, profile; minimum AGC target, 3.00e4; intensity threshold, 1.0e6; exclude isotopes, on; dynamic exclusion, 10.0 s. Each resuspended supernatant was consecutively injected and analyzed twice. Three nanograms of chemical standards were used to identify SAICAr (Musechem, R022231) and S-Ado (Wako Fujifilm, S688825) in separate runs.

MZmine2.53 (2) was used for baseline correction, mass detection, ADAP chromatogram building (3), chromatogram deconvolution, isotopic peaks grouping, feature list alignment, duplicate peak filtering, and gap filling. Raw data files were imported into MZmine2 without file format transformation. Ions eluted between 1 and 10 min were used for the following data pre-processing. Baseline correction was applied to unfragmented MS1 spectra: chromatogram type, TIC; m/z bin width, 0.1000; correction method, rolling ball baseline corrector. The mass was detected using the wavelet transform algorithm: noise level, 1.0E4 for MS1 and 5.0E3 for MS2; scale level, 5; wavelet window size, 30%. The peak model function “Lorentzian” was used for filtering shoulder peaks. Next, ADAP chromatogram builder was used for building MS1 chromatograms: min group size in # of scans, 5; group intensity threshold, 2.5E4; min highest intensity, 5.0E4; m/z tolerance, 0.001 m/z. The built MS1 chromatograms were then resolved into individual features by the *ADAP wavelets* algorithm using parameters as follows: S/N threshold, 10; S/N estimator, Intensity window SN; min feature height,

50,000; coefficient/area threshold, 70; peak duration range, 0.05 to 1; RT wavelet range, 0 to 0.05. Features corresponding to the ^{13}C isotopes of the same analyte were filtered out using the function *isotopic peaks grouper*: m/z tolerance, 0.001 m/z; retention time tolerance, 0.1 min; maximum charge, 2; representative isotope, most intense. Feature lists of different samples were then aligned using the *join aligner* function: m/z tolerance, 0.001 m/z; weight for m/z, 75; retention time tolerance, 0.1 min; weight for RT, 75; require same charge state, checked; compare isotope pattern, checked (isotope m/z tolerance, 0.001 m/z; minimum absolute intensity, 5.0E3; minimum score, 70%). Peaks detected within less than 70% of samples of the same tissue were filtered out. At last, gap-filling was performed using the *peak finder* algorithm to retrieve features that were not quantified in certain samples using parameters as follows: intensity tolerance, 50%, m/z tolerance, 0.001 m/z; retention time tolerance, 0.1 min; RT correction, checked. Peak areas of detected features were then exported as a primary result.

Data processing and quantitative analysis were done using R/RStudio (R4.3.2 and RStudio 2023.09.1+494). Features that were detected in more than 50% of pooled QC samples, reaching an RSD (relative standard deviation) value across pooled QC samples less than 30%, and having the average peak area across pooled QC samples larger than five times the one in the method controls were kept. For features of the same ions but detected in both positive and negative modes were recognized within an m/z difference of less than 2.014552 and a retention time difference of 0.1 min, values with the lowest RSD across pooled QC samples were kept in the dataset. Otherwise, features were removed from the dataset for statistical analysis. Missing values were imputed by the *missForest* R package. The drifts of MS-signal intensities were corrected based on the peak areas of the same feature over the pooled QC samples using the LOWESS software (http://prime.psc.riken.jp/compms/others/main.html#Lowess) with an automatically calculated optimal "Span" value. Peaks were normalized as previously described (upper-quartile normalization, 1). The normalized peak areas were then used for comparing the levels of SAICAr and S-Ado across genotypic and/or sex groups and statistical analysis. SAICAr was identified with m/z 375.115 and 243.072 for its precursor and fragment ions $[M+H]^+$ and peaking at the retention time of 6.1 min, and S-Ado was identified with m/z 384.115 and 252.072 for its precursor and fragment ions $[M+H]^+$ and peaking at the retention time of 6.7 min.

For the SAICAr concentrations, the values for some brain regions were averaged across subregions, specifically: measurements for the frontal pole and medial prefrontal cortex were combined for the frontal cortex; measurements from the motor, somatosensory, visual, entorhinal cortices were combined for the cerebral cortex; the dorsal and ventral hippocampi were combined for the hippocampus; the dorsal striatum and nucleus accumbens were combined for the striatum; the thalamus and hypothalamus were combined for diencephalon.

IntelliCage test paradigms

IntelliCage (TSE Systems GmbH, Bad Homburg, Germany) is a fully automated system as described (4, 5) with minor modifications. A 3D-printed adaptors were placed in front of the gates within each corner chamber to prevent mice from keeping the whole body within the corner, and the tunnel mouth of each corner chamber was covered by a 3D-printed ring to prevent it from being broken by the mice. The IntelliCage was placed in an isolation box, which is soundproof and has an independent ventilation/illumination system on a 12:12-h (6:00/18:00) light:dark cycle.

Prior to being introduced into the IntelliCage, a biocompatible glass-covered transponder (Ø2.12 × 12 mm) for RFID (radiofrequency identification) was subcutaneously implanted into adult hAdsl mice and WT littermates in an area below the ear under isoflurane inhalation anesthesia. The transponder chip allows the IntelliCage to identify the mouse who enters a corner chamber. After four days in their home cages, a handheld reader to confirm that the transponders were working before the mice (10 - 14 same-sex mice) were group-housed within a large plastic cage (TECNIPLAST, 61.2 × 43.5 × 21.6 cm^3) for one week. The numbers of mice in the cages were: Female: cage1, N_{WT}=5, N_{hAdsl} =8; cage2, N_{WT}=7, N_{hAdsl} =7; cage3, N_{WT}=7, N_{hAdsl} =7; cage4, N_{WT}=7, N_{hAdsl} =7; cage5, N_{WT}=7, N_{hAdsl} =6. Male: cage1, N_{WT}=5, N_{hAdsl} =6; cage2, N_{WT}=7, N_{hAdsl} =7; cage3, N_{WT}=7, N_{hAdsl} =7; cage4, N_{WT}=5, N_{hAdsl} =5; cage5, N_{WT}=7, N_{hAdsl} =7. Next, the IntelliCage system was introduced into the cage, and behavioral tests were conducted by the following procedures. Mice were adapted to the new system at the beginning three days (phase 1, "free adaptation") when the gates through which mice could access the water in all four

corner chambers were kept open, and thus, mice could drink water *ad libitum*. Following the free adaptation phase, all the gates in the four corner chambers were closed initially so that mice would be trained to open the gate and drink water using nose pokes in the coming four days (phase 1, "nose-poke adaptation"). The gate became open as soon as a nose poke was made by the mouse and closed five seconds later. Two or more nose pokes during the same visit couldn't open either gate again until the mouse exited and re-entered a corner chamber. Mice could access water with a nose poke for 24 hours in this phase.

A competitive task (phase 2) was then imposed on the mice. Mice were gradually deprived of water for 12 hours for 1 day, 16 hours for 1 day, 18 hours for 2 days, 20 hours for 1 day, and 21 hours for 9 days. On each day of this task, mice could access water at any one of the four corners starting from 10:00 pm (so-called "drinking session"). Mice could open any gate to drink water with a nose poke, and the gate was closed seven seconds later. The gate in this corner could be opened again until a different mouse visited it or until the same mouse exited and re-entered it. Two red LEDs in each of the four corners, one on each side, were switched on at the beginning of a drinking session and turned off when the session was over. In addition, an alert device (Tokyo Devices, Inc., Tsukuba, Japan) was attached to the side wall of the cage to notify the mice of the drinking session: a red LED was turned on and blinking for 10 seconds at the beginning of the drinking session; a buzzer was also beeping simultaneously for 10 seconds; the red LED then stayed on for the remaining session and was turned off when the session was over.

A second task with more intense competition followed in the following 13 – 14 days (phase 3). Mice were kept deprived of water for 21 hrs (drinking session: 10:00 pm – 1:00 am). In the first 6 – 7 drinking sessions, water was accessible at only one corner (rewarded corner) for all mice within the cage. The position of the rewarded corner was the same for all sessions. In the remaining sessions, the rewarded corner was changed to the diagonally opposite position, and its position was the same for all remaining sessions. Red LEDs were lit only in the rewarded corner, and not in the other three non-rewarded corners, during the drinking session to indicate the position. The alert device attached to the side wall of the cage was kept on as above to notify the mice of the drinking session.

IntelliCage data generated above, which was an array of RFID numbers, visit times, locations and the reward conditions for the visited corners, genotypes, sex, and experiment types, was analyzed using MATLAB (R2022b, MathWorks, California, USA). Null distributions for hypothesis tests were generated by random permutations of the genotypes within the cage. For each hypothesis test, 10,000 permutations were carried out.

Tube test

The tube test followed a previously published protocol (6). Briefly, 18- to 21-week-old male littermates and approximately 25-week-old female littermates were used. Naïve male mice, that had not been exposed to any other tests, were used. The female mice that underwent the tube test were first tested in the IntelliCage after which they rested for two weeks. Prior to the tube test, two hAdsl and two WT littermates of the same sex and similar weight (weight difference < 15%) and age were group-housed in a cage for two weeks. For the females, mice of the same IntelliCage were randomly group-housed with small weight differences (< 15%).

Three days before starting the tube test, mice were handled and freely explored the workbench for one to two min to reduce their stress. The tail of each mouse was uniquely marked using a marker pen. Mice were taken from the holding room to the behavioral test room for habituation for at least 20 min before starting the training and tube tests. Each mouse was trained to move forward out of a transparent acrylic tube (MiSuMi: outer and inner diameters, 35 and 29 mm; length, 30 cm) five times from alternating ends of the tube for 2 days. If the mouse retreated or stopped moving in the tube for more than 2 min, it was gently pushed by touching its back with a cotton swab stick. The tube and workbench surface were cleaned between trials with 70% ethanol and dried with tissue paper. Next, mice were tested daily for at least seven days and until reaching a stable rank in three consecutive days. Before starting each of the test trials, each mouse was trained again to move out of the tube once from each end. During the test trial, six unique pairs of mice within a cage were tested. The paired mice were released simultaneously into the opposite ends and met around the middle of the tube. The mouse that

first retreated from the tube was designated as the "loser", while the mouse that was still in the tube was designated as the "winner". The tube and workbench surface were cleaned between each trial with 70% ethanol and dried with tissue paper. From trial to trial, the same mouse was released into the tube from alternative ends. The order of paired mice in each trial was randomized daily. The number of wins of each mouse, which varied from 0 to 3, was calculated as an index of social dominance. The four mice within a cage were ranked from 1 to 4 based on their indices of social dominance.

Muscle performance tests

Mice of both sexes and genotypes, between 8 and 12 weeks old, were used for behavior tests assessing muscle strength and running ability. Three tests evaluating muscle strength, including the grip strength test, weights test, and wire-hanging test, and the sprint and endurance tests using a treadmill were adapted from established protocols (7-11) for measuring rodent grip strength with some modifications. All devices and the working bench surface were cleaned with 70% ethanol and dried between trials/sessions.

Grip strength test

The mouse was held by the base of the tail and lowered over the grid, with its torso remaining horizontal and only the forepaws contacting the grid of a grip strength meter (MK-380Si, Muromachi Kikai Co., Ltd., Tokyo, Japan). Then the mouse was gently pulled away from the grid horizontally, during which the maximal grip strength was recorded from the meter's display. This process was repeated twice more, yielding three trials in total. The average forelimb grip force of the mouse was then calculated and normalized against its body weight.

Weights test

A series of weights were made from tangled fine gauge stainless steel wire attached to steel chain links, ranging from 20 g to 100 g. The mouse was held by the tail and lowered to grab the weight placed on the bench, starting with the lightest. Once the mouse grabbed the wire ball with its forepaws, the experimenter gently raised it until the chain link left the bench surface. The mouse must hold the weight for at least 3 seconds to proceed to a heavier weight, and the holding duration for the successful lift was recorded as 3 seconds. The hold duration was recorded if the mouse dropped the weight before 3 seconds. Then after a brief 10-second rest, the mouse was held to attempt the same weight again; if it failed to hold the weight for three consecutive attempts, the trial concluded, recording the maximum weight and the maximum holding duration for the maximum weight achieved. A final score is calculated as the sum of the time holding each weight.

Wire-hanging test

A 90 cm-long metal wire with a diameter of 2 mm was bent and connected into a circle and suspended 90 cm above a padded cage. The mouse's forepaws were placed on the bottom of the loop. A stopwatch was started once the mouse grasped the wire, and the latency until it fell was recorded. The mouse was removed from the loop when a maximum time of 2 minutes was reached. This procedure was repeated three times with a one-minute recovery period between each trial, and the average latency across the trials was calculated.

Running ability on the treadmill

Mice were recruited for treadmill training and tested between 8 - 10 weeks old. Each mouse was placed into individual lanes on a treadmill (model 76-0896, Panlab Harvard Apparatus, Holliston, MA, USA) set on a flat surface at a 10° incline. The treadmill was initially off, then after allowing the mice to explore in their lane for 5 minutes freely, the speed was gradually increased to 3.0 m/min in 5 minutes. The experimenter observed and ensured all mice started walking using gentle taps with a tongue depressor to encourage movement. After that, the speed was increased from 5 to 7, 10, 12, and 15 m/min

in 5-minute intervals. The training was conducted over three successive days to ensure familiarization with the treadmill.

After the three-day training, mice were tested for running ability. For testing the endurance, mice were placed into the lanes when the treadmill was off; then the treadmill was set to 12 m/min at a 15° incline with an initial speed of 12 m/min for 30 seconds. Then, the speed was gradually increased with the following steps: 14 m/min for 30 seconds, 16 m/min for 5 minutes, 18 m/min for 24 minutes, and then a 2 m/min increase every 15 minutes until reaching 26 m/min. If the mouse stayed in a region of one body length encompassing the electric shock grid at the end of the treadmill belt for five continuous seconds or longer, it is considered as reaching fatigue and removed from the treadmill. The total running time and distance were recorded for each mouse, and the session concluded once all mice were removed from the treadmill. As for the sprint test, mice were allowed to warm up at 6 m/min with a 15° incline for 5 minutes, and then the treadmill was operated following the subsequent steps: (1) increasing the speed to 14 m/min for 30 seconds, followed by a 1.5-minute run at 6 m/min; (2) increasing the speed to 18 m/min for 30 seconds, followed by another 1.5-minute run at 6 m/min; (3) increasing the speed from 18 to 20, 22, and 24 m/min with 15-second intervals, followed by a 3-minute run at 6 m/min; (4) increasing the speed from 22 to 26, 30, 34 m/min with 15-second intervals, followed by another 3-minute run at 6 m/min; and (5) repeating the same pattern for maximal two more rounds with increasing speed until the mice could not keep pace and stayed to the grid behind the treadmill. The mice that stayed on the grid for 5 seconds or longer were removed from the treadmill, and their maximum speed achieved was recorded.

mRNA expression

The bulk tissue gene expression for human ADSL (ENSG00000239900.12) was retrieved from the GTEx portal "Expression" (dbGaP accession number phs000424.v8.p2, https://gtexportal.org/home).

To estimate the mRNA expression of the Adsl gene in mouse tissues, the gene read counts of publicly available RNA sequencing (RNA-seq) datasets were obtained from the Gene Expression Omnibus

(GEO): GSE194203 (brain and non-brain tissues) (12) and GSE178290 (male brains) (13). The gene read counts of GEO datasets were normalized into TPM values for comparisons in R/RStudio. Gene length information was retrieved from the NCBI RefSeq assembly GCF_000001635.27 (https://ftp.ncbi.nlm.nih.gov/genomes/all/GCF/000/001/635/GCF_000001635.27_GRCm39/). The Pearson correlation of gene expression and fold metabolic change was performed using the *stat_cor()* function in an R package "ggpubr".

To quantify *Adsl* mRNA expression in the brain of hAdsl and WT littermates, three anatomical regions (amygdala, visual cortex, and cerebellar cortex) were dissected from both male (N_{WT}=7, N_{hAdsl}=7) and female (N_{WT}=5, N_{hAdsl}=8) mice on dry ice to prevent thawing. Total RNA was extracted using the Monarch® Total RNA Miniprep Kit (New England BioLabs, T2010S) and quantified with the NanoDrop2000c spectrophotometer. For cDNA synthesis, 150 ng of total RNA and random hexamers were used in 10 µl reaction volumes with the PrimeScrip™ RT Reagent Kit (Perfect Real Time) (Takara, RR037A). Quantitative PCR (qPCR) was performed using the Applied Biosystems StepOnePlus qPCR system with the Luna® Universal qPCR Master Mix (New England BioLabs, M3003S). Expression levels were quantified using the ΔCt method, with mouse *Gapdh* serving for normalizing mRNA expression, and four (*Gapdh*) to five (*Adsl*) technical replicates were conducted for each gene. The primer sequences used were as follows: Mouse *Adsl*, 5'-TGTCGGAGACAATACGGACC-3' (forward), 5'- TTTACTCCTCGGAAGCGCAG-3' (reverse); Mouse *Gapdh*, 5'- TGTGTCCGTCGTGGATCTGA-3' (forward), 5'-CCTGCTTCACCACCTTCTTGA-3' (reverse).

For bulk RNA sequencing of skeletal muscles, the gastrocnemius, tibialis anterior, and soleus muscles were dissected from the hindlimbs (N = 3 per sex per genotype) and immediately snap-frozen in liquid nitrogen. RNA extraction, library preparation, and sequencing were conducted at the OIST Sequencing Center, and the raw data are available via the Gene Expression Omnibus (GEO) repository (BioProject accession number: PRJNA1272784). Briefly, the snap-frozen muscle samples were pulverized using a Freeze Crusher µT-48 (TAITEC Corporation) for a total of 2 minutes with 30-second intervals. The powdered tissues were then homogenized in lysis buffer containing 2-Mercaptoethanol

using a Polytron PT 1200 E homogenizer. Total RNA was isolated from the resulting homogenates using a KingFisher Apex Machine (Thermo Fisher Scientific Inc.) with the MagMAX mirVana Total RNA Kit (Thermo Fisher Scientific Inc.). Paired-end sequencing (250 bp) was performed on an Illumina NovaSeq 6000 platform. Raw reads underwent quality control using Fastp (v0.23.2) and were aligned to the reference genome (GRCm39, GCA_000001635.9) using STAR (v2.7.11a). The aligned reads were further assembled and quantified using StringTie (v2.2.1) for transcript assembly and quantification, with transcript abundances reported as transcripts per million (TPM).

Immunoblotting

Total proteins from snap-frozen muscles (30 mg of gastrocnemius collected from each mouse) were extracted using the Minute Total Protein Extraction Kit for Muscles (Invent Biotechnology) following the manufacturer's instructions. The extracts were stored at -80°C before the subsequent experiments. Protein concentrations were determined by Pierce BCA assay. Then for each sample, the total protein was reduced and loaded (6 µg/lane), separated in a 4-12% Bolt Bis-Tris Plus Mini Protein Gel (Invitrogen, ThermoFisher Scientific), and transferred to polyvinylidene fluoride (PVDF) membranes. The membranes were blocked with Bullet Blocking One (Nacalai Tesque, Kyoto, Japan), and then probed with the primary antibodies (ADSL polyclonal antibody, 1:2,000, PA5-29964, Invitrogen, ThermoFisher Scientific; anti-GAPDH monoclonal antibody, 1:10,000, 016-25523, FUJIFILM Wako Pure Chemical Corporation, Japan) at 4°C overnight, followed by 1-hour incubation of the secondary antibodies (Anti-Rabbit IgG(Goat), HRP-conjugated, Pre-absorbed, 1:10,000, 21858-24, Nacalai Tesque; Goat anti-Mouse IgG (H+L) Highly Cross-Adsorbed Secondary Antibody, Alexa Fluor™ 647, 1:10,000, Invitrogen, ThermoFisher Scientific). After washing, the membranes were incubated with a chemiluminescence substrate (Chemi-Lumi One L, Nacalai Tesque) and imaged using the ChemiDoc MP imaging system (Bio-Rad). The band intensities were quantified using ImageJ/Fiji.

Tajima's D analysis

The phased haplotypes data, consisting of a harmonized dataset of 4091 human genomes from the 1000 Genomes Project (1kGP) and Human Genome Diversity Project (HGDP), was downloaded from gnomAD (14, gs://gcp-public-data - - gnomad/resources/hgdp_ 1kg/phased_haplotypes_v2/). This dataset includes individuals from seven super-populations: Africa (AFR, 986 individuals), America (AMR, 549 individuals), Central South Asia (CSA, 782 individuals), East Asia (EAS, 816 individuals), Europe (EUR, 771 individuals), Middle East (MID, 157 individuals), and Oceania (OCE, 30 individuals). Tajima's D values were calculated using VCF-kit (15) with a sliding window of 25,000 bp and a step size of 1 bp.

Analysis of Ancestral Recombination Graphs

Tests for positive selection were also used Ancestral Recombination Graphs (ARGs) and analyzed them with CLUES2. ARGs were inferred using ARGweaver-D (16) from phased genotype data for 18 present-day individuals from the 1000 Genomes Project (6 African, 6 European, and 6 East Asian, randomly selected from each continental population group), along with the Altai, Chagyrskaya, and Vindija Neandertal and Denisovan genomes. All data were mapped to the GRCh37 (hg19) reference human genome. Trees were extracted from 5,000 MCMC iterations after a burn-in of 2,000 iterations, and local genealogies at the SNP positions were analyzed using CLUES2 (17), which estimates a selection coefficient for a derived allele after applying importance sampling to the ARGs sampled by ARGweaver-D. CLUES2 was run with the options `--popFreq 0.95 --N 20000 --tCutoff 200000`. Based on linkage disequilibrium calculated in PLINK v1.90b7, the variants were grouped into two haplotypes for separate analysis: one including rs12160297, rs56362590, and rs61704599, and another corresponding to rs8135371.

To generate a control set with similar allele frequencies to *ADSL*, 22 variants were selected with ≥95% homozygous reference alleles in modern humans and 100% homozygous alternate genotypes in archaic individuals. These variants were filtered to retain only biallelic SNPs in intergenic regions that

were classified as benign in ClinVar. Variants were excluded if they were located within 100 kb of annotated genes or overlapping conserved sites based on PhyloP, PhastCons, or GERP++ scores, and it was required that the selected sites were at least 35 kb apart. Genealogies for these sites were inferred using ARGweaver-D in 1 Mb windows centered on each control variant using the same panel of individuals as for *ADSL*. CLUES2 was then applied to the sampled genealogies to generate empirical null distributions for selection coefficients and p-values, against which the results for *ADSL* were compared.

Molecular Biology

Wild-type human adenosine A1, A2A, A2B, and A3 receptors and G-protein coupled inward-rectifying potassium channels 1 and 4 (GIRK1 and GIRK4) cDNA were subcloned into the oocyte-adapted pXOOM vector (18, Genscript, NJ, USA). The sequences were verified by sequencing. Plasmids were linearized using appropriate restriction enzymes followed by in vitro transcription using the T7 mMessage mMachine kit (Ambion, TX, USA). cRNA concentration and purity were determined by spectrophotometry.

Oocyte Preparation

Oocytes from the African clawed toad, *Xenopus laevis*, were provided by Ecocyte Biosciences (Dortmund, Germany). Following one day of incubation at 12 °C in modified Barth's solution (19), oocytes were injected with 0.2 ng A1/A2A/A2B/A3 receptor cRNA and 40 pg of each GIRK1 and GIRK4 cRNA using the Nanoject III (Drummond Scientific, PA, USA). The volume was 50 nl per oocyte.

Ligands

Adenosine, S-Ado, and SAICAR were purchased from MedChemExpress (NJ, USA). Adenosine was dissolved into recording buffer, whereas S-Ado and SAICAR were dissolved in DMSO (Sigma-Aldrich, MO, USA) and further diluted in recording buffer. The maximum final DMSO concentration was 0.3% v/v.

Electrophysiology.

RNA-injected oocytes were incubated at 12 °C for six days prior to electrophysiological experiments, which were performed using the parallel eight-channel, semi-automated, two-electrode voltage-clamp OpusXpress 6000A (Molecular Devices, CA, USA) (19). Data were acquired at a membrane potential of −80 mV and sampled at 156 Hz using OpusXpress 1.10.42 (Molecular Devices) software. To increase the GIRK1/4 currents at negative potentials, a high potassium concentration extracellular recording buffer was used (64 mM NaCl, 25 mM KCl, 0.8 mM $MgCl_2$, 0.4 mM $CaCl_2$, 15 mM HEPES, adjusted to pH 7.4 with NaOH), yielding a K+ reversal potential of about −40 mV. Oocytes were perfused with buffer at 1 ml/min. Ligand concentrations higher than 30 μM were not tested to avoid effects of DMSO used to dissolve the compounds. In preliminary experiments, the A2B receptor showed very low adenosine potency in our system and was excluded from further work. For each oocyte, the GIRK1/4 response to a given concentration of agonist was normalized to the response to the highest concentration of adenosine tested. Electrophysiological data were analyzed in Clampfit 10.6 (Molecular Devices). Concentration–response curves were calculated using a four-parameter sigmoidal function in GraphPad Prism 10 (GraphPad Software, CA, USA), with unconstrained parameters, except for the Hill slope (constrained to 1.0).

Suppl. Fig. 1.

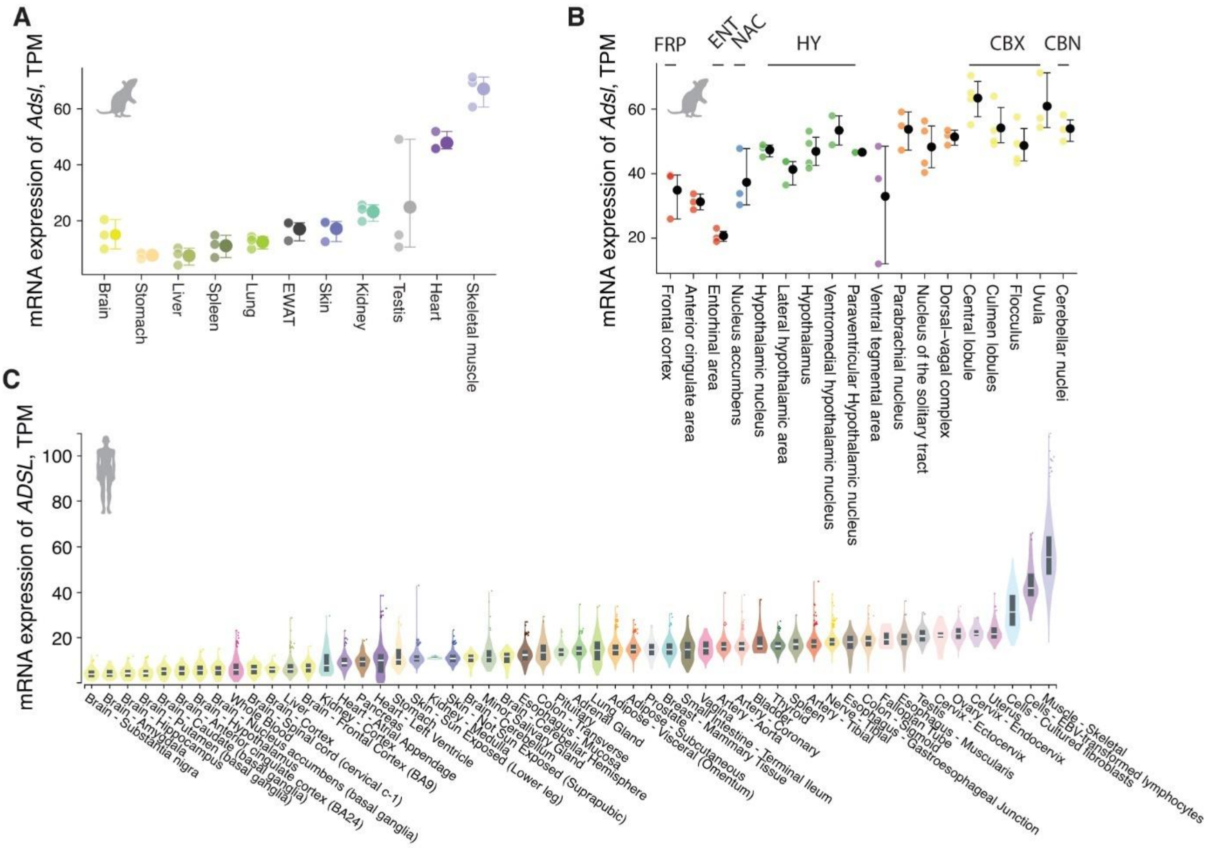

Suppl. Figure 1. ***ADSL* expression in mice and humans. (A)** *Adsl* mRNA expression in mouse tissues. Data are presented as mean ± 95% CI. TPM: transcripts per million. EWAT: Epididymal white adipose tissue. **(B)** The levels of *Adsl* mRNA expression in brain regions of mice. HY: hypothalamus; CBX: cerebellar cortex; CBN: cerebellar nuclei; FRP: frontal pole; ENT: entorhinal cortex; NAc: Nucleus accumbens. **(C)** *ADSL* mRNA (ENSG00000239900.12) in human tissues. Data are extracted from the GTEx Portal [GTEx Analysis Release V8 (dbGaP Accession phs000424.v8.p2)].

Suppl. Fig. 2.

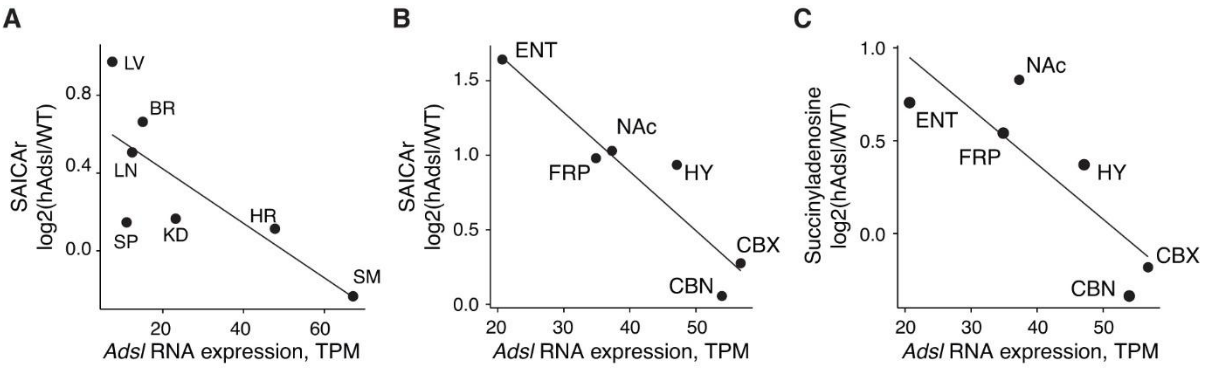

Suppl. Figure 2. Correlations of *Adsl* expression and SAICAr and syccinyladenosine fold-changes in the mouse brain. (A) Correlation of SAICAr fold-changes in the brain and non-brain tissues of hAdsl mice and *Adsl* mRNA expression in tissues of wild type mice in an RNA sequencing (RNAseq) data in the Gene Expression Omnibus (GEO): GSE194203 (also see Fig. S1A). SAICAr fold-change in the brain is an average of the fold-changes in cerebrum and cerebellum. BR, brain; HR, heart; LN, lung; LV, liver; KD, kidney; SP, spleen; SM, skeletal muscle. Pearson correlation: R = -0.78, p = 0.04. **(B)** Correlation of SAICAr fold-changes in brain regions of male hAdsl mice and *Adsl* mRNA expression in wild-type males in GEO: GSE178290. Pearson correlation: R = -0.94, p = 0.006. FRP, frontal pole; NAc, nucleus accumbens; ENT, entorhinal cortex; HY, hippocampus; CBX, cerebellar cortex; CBN, cerebellar nuclei. **(C)** Correlation of succinyladenosine fold-changes in the brain of male hAdsl mice and *Adsl* mRNA expression in the brain of wild-type males in an RNAseq data set deposited in GEO: GSE178290. Pearson correlation: R = -0.84, p = 0.036.

Suppl. Fig. 3.

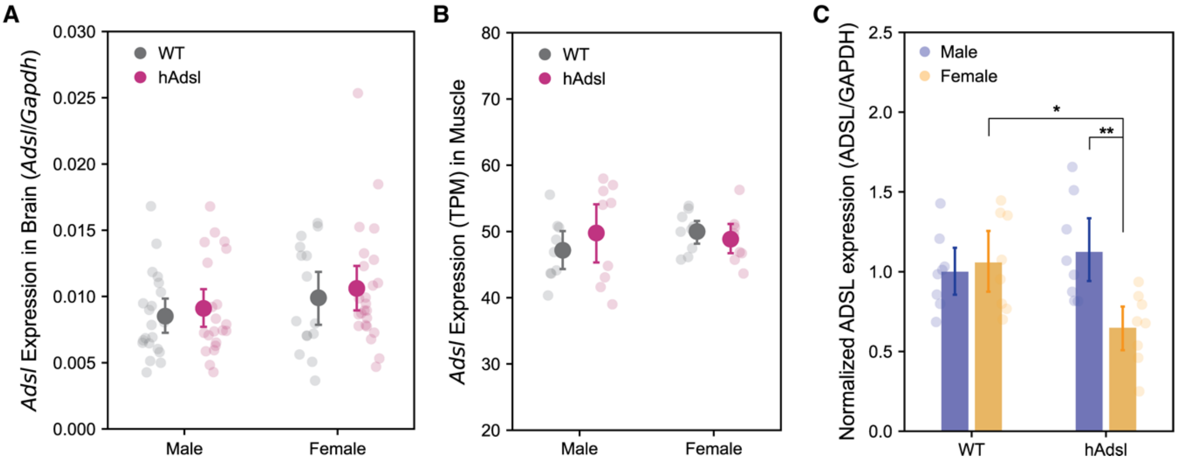

Suppl. Figure 3. ADSL mRNA and protein in WT and hAdsl mouse brain and muscle. (A) *Adsl* mRNA levels in the brain detected by qPCR. **(B)** *Adsl* mRNA levels in skeletal muscles (tibia anterior, soleus, and gastrocnemius muscles) measured by bulk RNA-seq. (**C**) The amounts of ADSL proteins in the gastrocnemius muscle detected by immunoblotting, normalized to GAPDH as a loading control, and to the expression of wild type males. In humanized mice, females express 42% less ADSL protein than males. Data are presented as mean ± 95% CI; Two-way ANOVA (post-hoc Tukey's Honest Significant Difference test), *: p-value < 0.05, **: p-value < 0.01.

Suppl. Fig. 4.

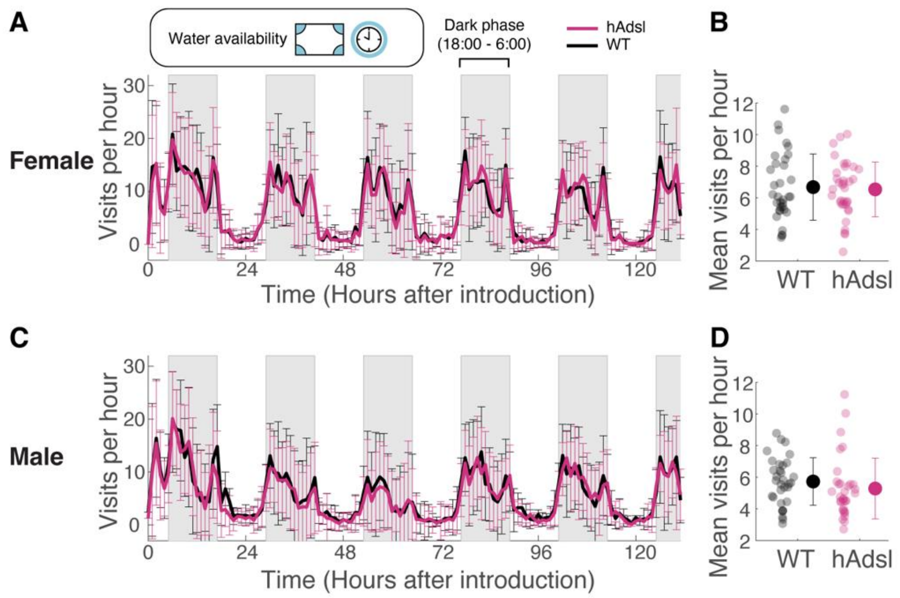

Suppl. Figure 4. hAdsl mice and WT littermates do not differ in corner visiting during spontaneous exploration. (A) Hourly visit frequencies to any of the four corners by individual female mice after introduction to the IntelliCage. Thick lines are averages over individual mice (magenta = 35 humanized Adsl mice; black = 33 WT mice, 5 cages). Water was available for 24 hours at all corners. **(B)** Mean hourly visit frequencies over 6 days of female hAdsl and WT littermate individuals. Summarized data are presented as mean ± standard deviation (error bars). **(C)** The same as in (A) for male mice. N = 33 humanized Adsl mice and 32 WT mice from 5 cages. **(D)** Mean hourly visit frequencies over 6 days of male hAdsl and WT littermate individuals. Summarized data are presented as mean ± standard deviation (error bars).

Suppl. Fig. 5.

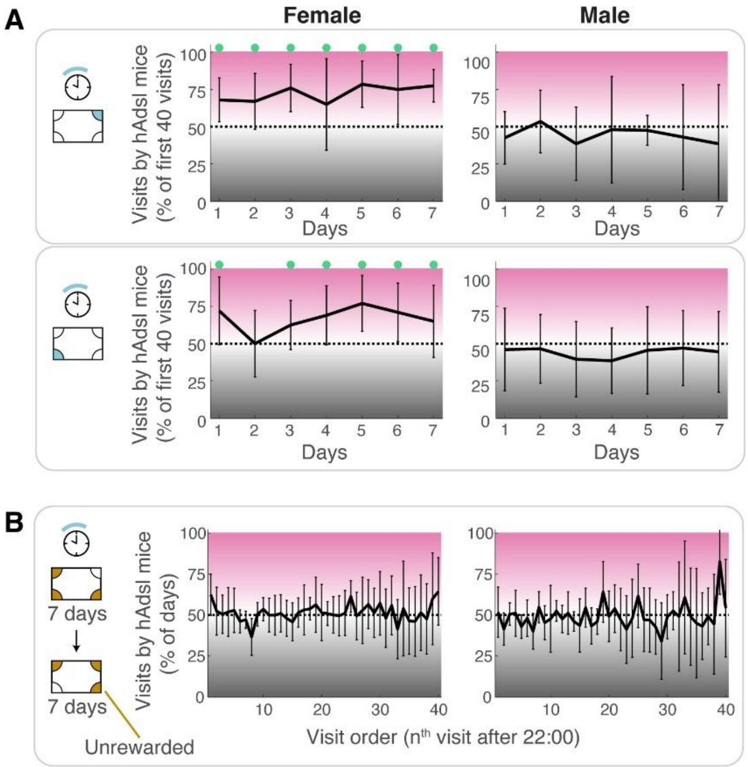

Suppl. Figure 5. Female hAdsl mice access water more persistently when water availability is restricted to only one corner. (A) The proportion of hAdsl mice among the first 40 visits after 22:00 for each corner on each day. Water was available at "corner 2" for the first 7 days and switched to "corner 4" for the subsequent 7 days. Left and right panels show female and male cages, respectively (5 each). Means ± standard deviation across cages are shown. Green dots indicate a significant difference from shuffled datasets at $p = 0.05$ level (two-tailed test with Bonferroni correction). Dotted horizontal lines at 50% indicate equal access by hAdsl and WT mice. **(B)** The proportion of days on which hAdsl mice were observed at the specified, early visit positions in unrewarded corners, as indicated in the schematic diagram, after 22:00. The total number of days analyzed was 14 days for each cage.

Suppl. Fig. 6.

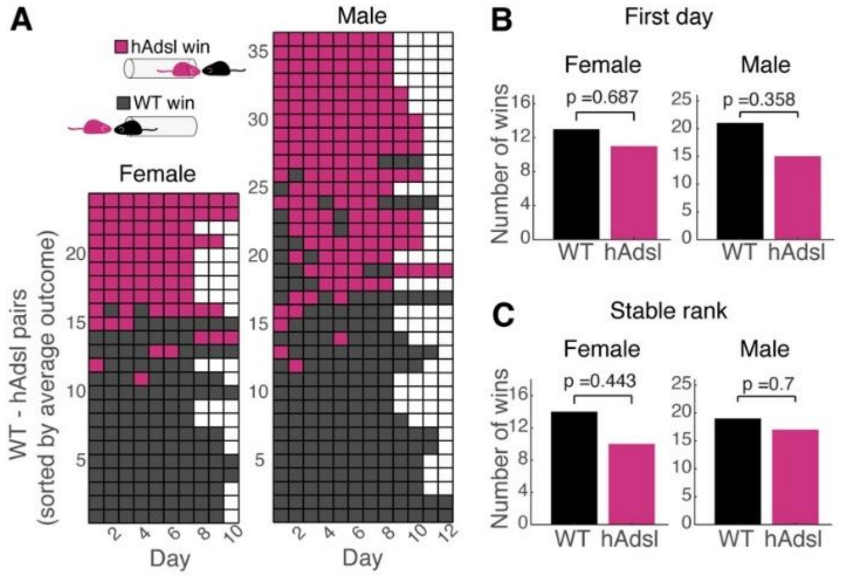

Suppl. Figure 6. hAdsl mice and WT littermates do not differ in tube tests. (A) Outcomes of individual pairing is color-coded (magenta = hAdsl mouse won; black = WT mouse won). White indicates that no encounter took place. **(B)** Summary of outcomes from the first day, showing the total number of wins made by WT mice and hAdsl mice. **(C)** Mice were ranked in each home cage based on the number of wins. When the ranks did not change over three consecutive days, the ranks were considered stable. Summary of outcomes from the last day, when stable ranks have been established, showing the total number of wins made by WT mice and hAdsl mice. P-values are for two-tailed tests against shuffled controls repeated 10,000 times.

Suppl. Fig. 7.

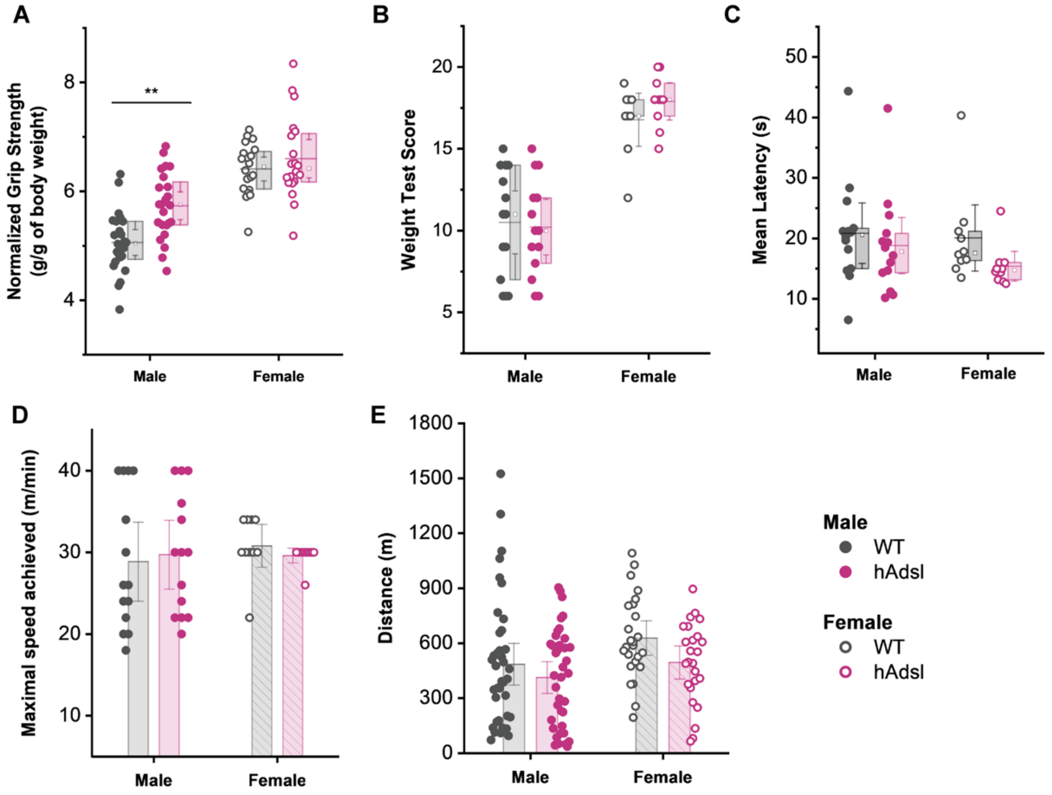

Suppl. Figure 7. Muscle strength and running ability of hAdsl mice and their WT littermates. Three tests of muscle strength and two running tests on the treadmill were used to evaluate muscle performance. **(A)** Females had higher normalized grip strength than males and hAdsl males higher grip strength than WT males (male: N=24 per genotype; female: N=20 per genotype). **(B)** Females performed better than males in weight lifting; no difference was observed between genotypes (male: N=14 per genotype; female: N_{WT}=9, N_{hAdsl}=10). **(C)** In a wire-hanging test, mice of both sexes and genotypes performed similarly (male: N=14 per genotype; female: N=10 per genotype). In running tests, no differences were observed between sexes or genotypes in **(D)** endurance (male: N_{WT}=39, N_{hAdsl}=38; female: N=25 per genotype) and **(E)** sprinting (male: N=14 per genotype, female: N=10 per genotype). Data are presented as mean ± 95% CI; Two-way ANOVA (post-hoc Tukey's Honest Significant Difference test), **: $p < 0.01$.

Suppl. Fig. 8

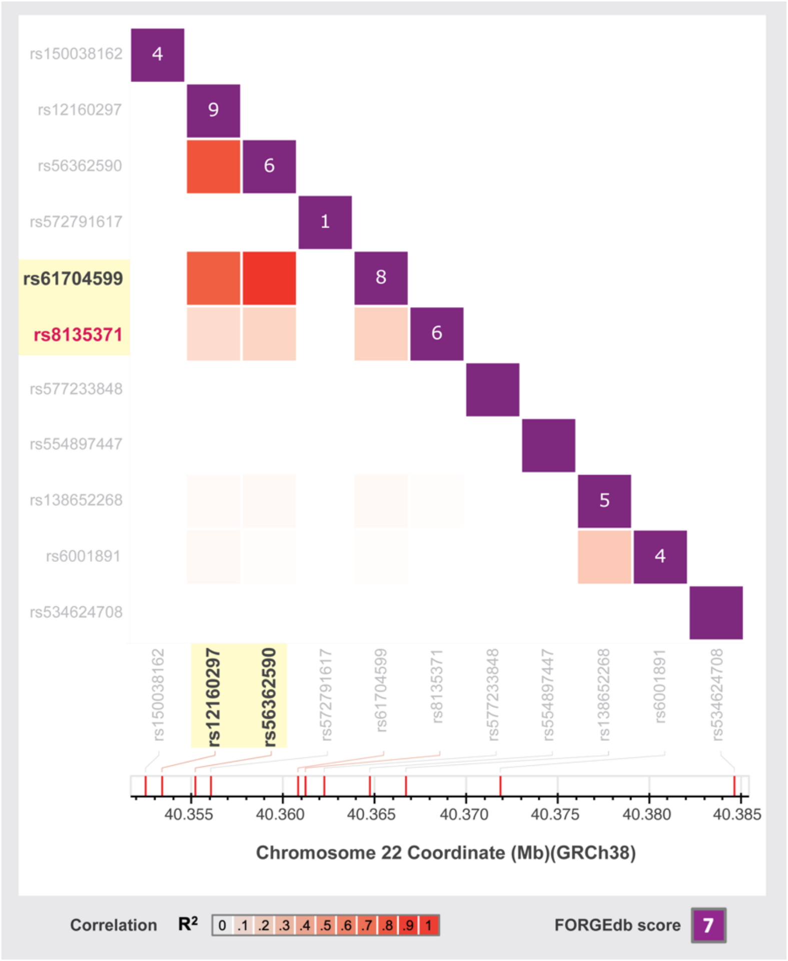

Suppl. Figure 8. A haplotype associated with lower *ADSL* expression in present-day humans. Eleven derived variants in the ADSL gene have risen to frequencies >80% in humans. Four of them (yellow-shadowed IDs) form a haplotype. The high FORGEdb scores (https://forgedb.cancer.gov/) evaluates the likely functional importance of genetic variants on a scale from 0 to 10.

Suppl. Fig. 9.

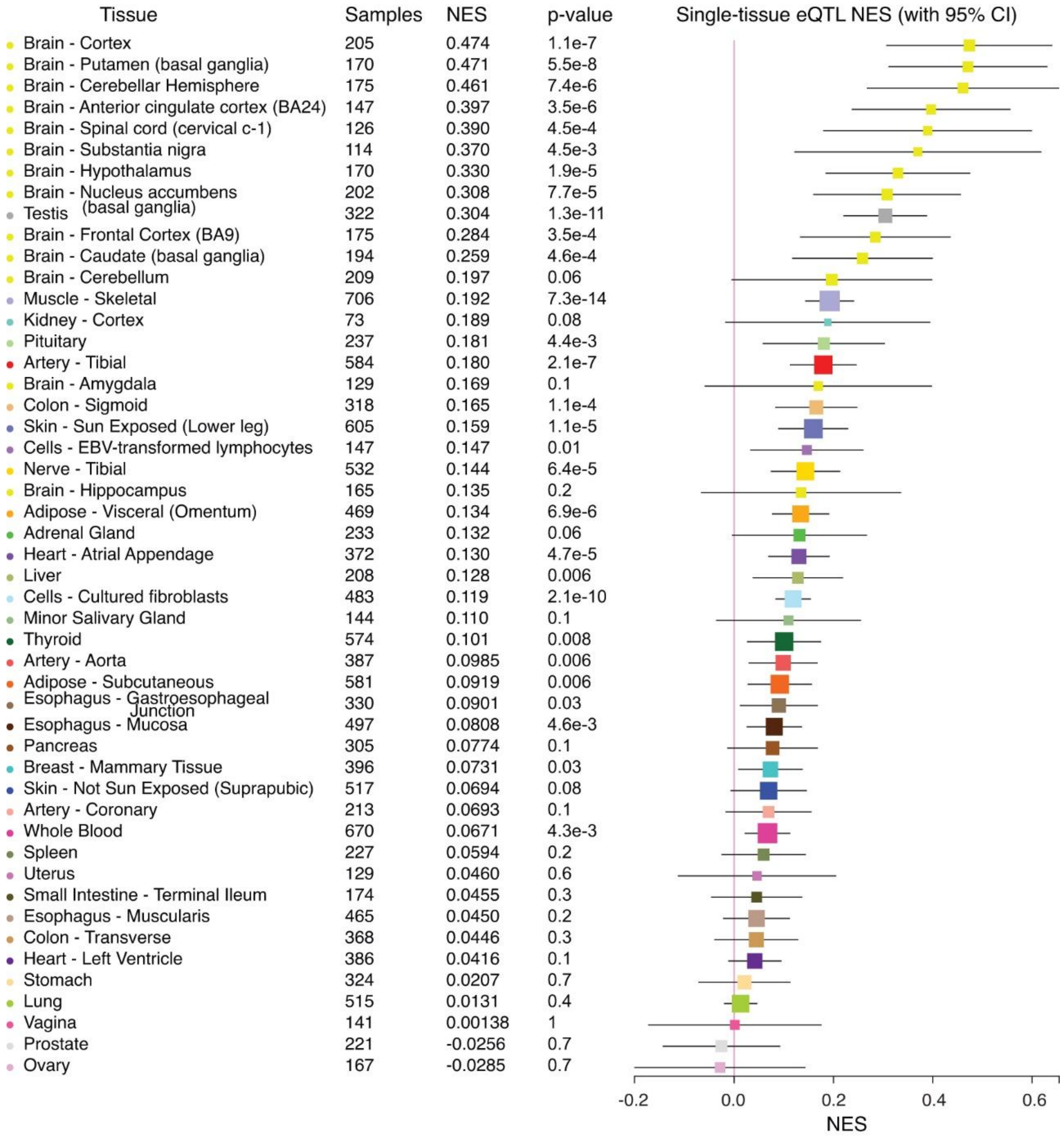

Suppl. Figure 9. rs8135371 as an expression quantitative trait loci (eQTL) for *ADSL*. The effect of variant C relative to variant A on the mRNA expression of the *ADSL* gene (ENSG00000239900) in 47 human tissues and cells. Data from the Genotype-Tissue Expression (GTEx, Release V8) project. NES: normalized effect size.

Suppl. Fig. 10.

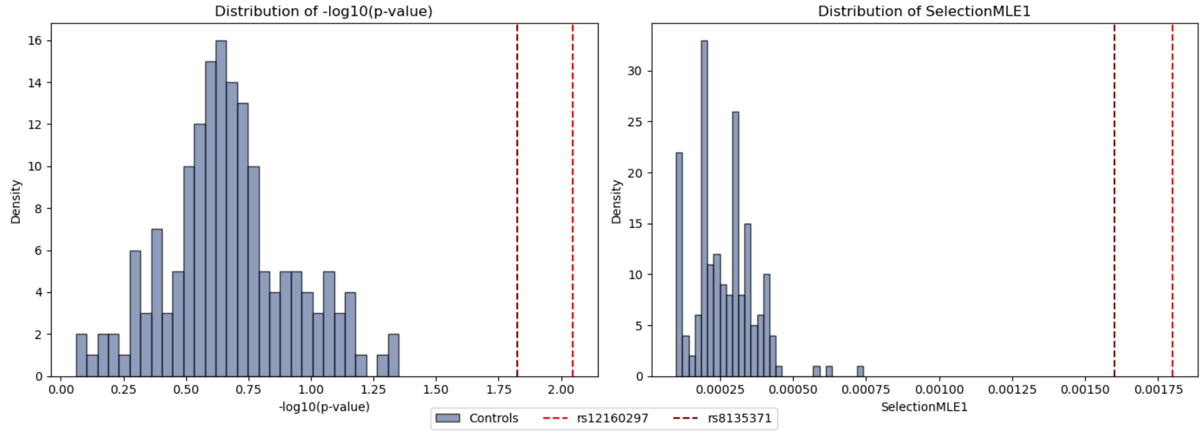

Suppl. Figure 10. Estimated –log p-values and selection coefficients from the ARG-based analysis of ADSL. Estimates were obtained using CLUES2 on ARGs sampled with ARGweaver-D. Results are shown for two haplotypes, tagged by SNPs rs12160297 (vertical dashed red line) and rs8135371 (vertical dashed dark red line), in comparison to an empirical null distribution (in blue) based on 188 noncoding loci with similar allele frequencies (see Methods).

Suppl. Fig. 11.

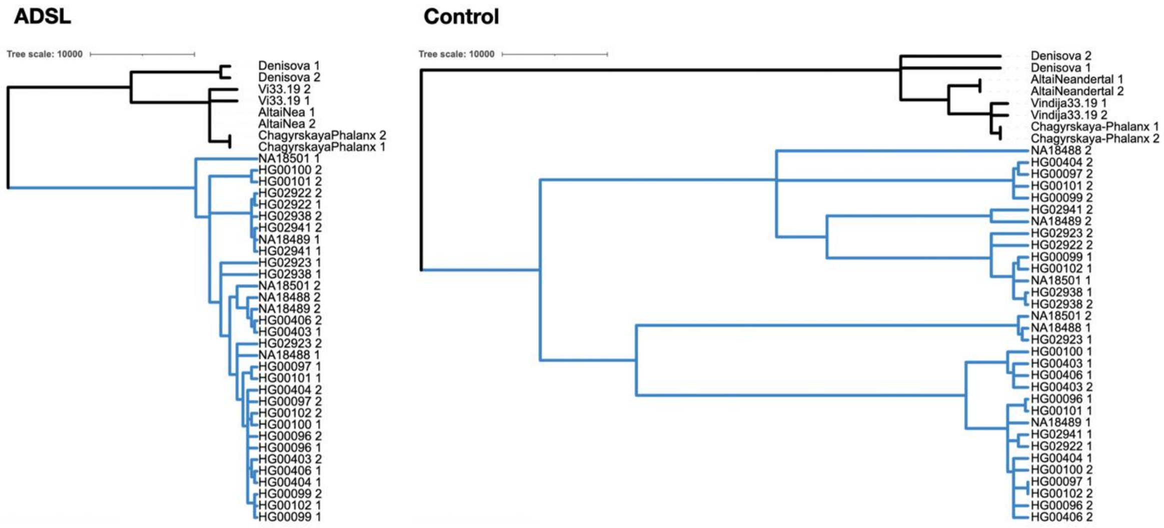

Clues2 results

Region	logLR	-log10(p-value)	SelectionMLE1	p-value
Control	**0.3569**	0.40	0.00020	0.398
ADSL	**3.3871**	2.03	0.00179	0.009

TMRCA comparison

Tree	Modern	Archaic	Modern/Archaic
Control	46398	15576	2.97
ADSL	5885	10176	0.57

Suppl. Figure 11. Representative genealogies for ADSL and a control locus. Genealogies were sampled by ARGweaver-D at the haplotype tagged by SNP rs12160297 in ADSL (left) and for one of the 22 matched controls (right). The ADSL genealogy exhibits a burst of coalescence events following the diversification of modern human lineages, consistent with a rapid rise in frequency of a selectively favored allele. By contrast, the coalescence events are more dispersed for the control locus, and the time to most recent common ancestor is much greater. This is the pattern that CLUES2 uses in estimating selection coefficients for *ADSL*.

Suppl. Fig. 12.

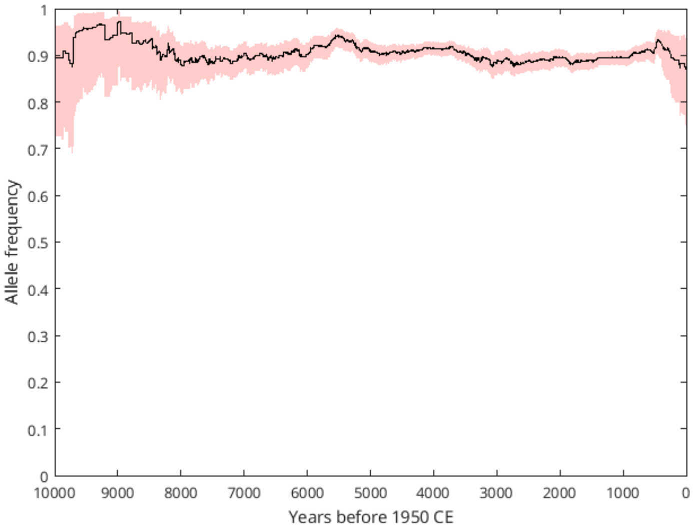

Suppl. Figure 12. Allele frequencies of the rs8135371-A variant over the past 10,000 years in Europe. Allele frequencies were calculated from 3,865 ancient European genomes, using a sliding 1,000-year window and 1-year step intervals. The shaded area represents the 95% confidence interval, calculated using Wilson score intervals. All genomes analyzed are pseudo-haploid, with data sourced from the Allen Ancient DNA Resource, version 54.

Suppl. Fig. 13.

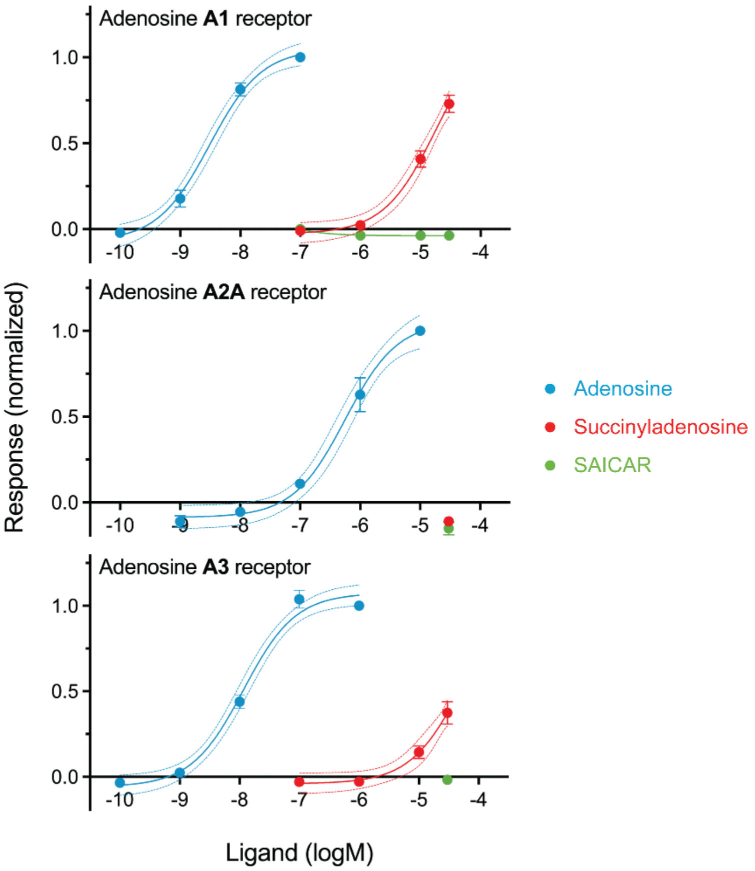

Suppl. Figure 13. SAICAR and succinyladenosine do not activate adenosine A1, A2A and A3 receptors. Adenosine A1, A2A, and A3 receptors were co-expressed with G protein-coupled inward-rectifying potassium channels in *Xenopus* oocytes. The panels give concentration-response data for the A1 (upper), A2A (middle), and A3 receptors (lower), respectively, for adenosine (blue), SAICAR (green) and succinyladenosine (red). The latter compound preparation contains 0.026% adenosine, explaining the observed response at micromolar succinyladenosine concentrations. 95% confidence intervals are shown. For each receptor, the responses are normalized to the response to the highest adenosine concentration tested.

Suppl. Table 1. Levels of SAICAr and S-Ado in seven tissues of hAdsl and WT littermate mice, sexes separated.

Sex	Tissue abbr.	Tissue	Mean (SAICAr in hAdsl)	Mean (SAICAr in WT)	Mean (S-Ado in hAdsl)	Mean (S-Ado in WT)	Fold change of SAICAr (hAdsl vs WT)	SAICAr_p.value (hAdsl vs WT)	Fold change of S-Ado (hAdsl vs WT)	S-Ado_p.value (hAdsl vs WT)
Female	HR	Heart	0.61	0.50	0.65	0.56	1.24	0.41	1.17	0.54
Male	HR	Heart	0.72	0.77	0.78	0.82	0.94	0.71	0.95	0.87
Female	KD	Kidney	0.75	0.70	0.47	0.77	1.07	0.8	0.61	0.34
Male	KD	Kidney	0.53	0.45	0.50	0.58	1.18	0.47	0.86	0.67
Female	LN	Lung	0.71	0.48	0.78	0.54	1.48	0.03	1.45	0.03
Male	LN	Lung	0.76	0.55	0.87	0.68	1.37	0.04	1.28	0.21
Female	LV	Liver	1.21	0.60	0.81	0.79	2.01	0.000008	1.03	0.92
Male	LV	Liver	0.74	0.39	0.70	0.83	1.91	0.00005	0.85	0.32
Female	SM	Skeletal muscle	0.83	1.25	0.82	0.56	0.66	0.34	1.47	0.33
Male	SM	Skeletal muscle	0.64	0.59	0.36	0.94	1.09	0.73	0.38	0.36
Female	SP	Spleen	0.76	0.57	0.60	0.82	1.34	0.08	0.73	0.38
Male	SP	Spleen	0.57	0.62	0.42	0.64	0.91	0.7	0.66	0.19
Female	CR	Cerebrum	1.92	0.99	1.71	1.58	1.93	0.0005	1.08	0.56
Male	CR	Cerebrum	1.41	0.65	1.47	1.40	2.18	0.00003	1.05	0.72

Suppl. Table 2. Levels of SAICAr and S-Ado in seven tissues of hAdsl and WT littermate mice, sexes combined.

Tissue abbr.	Tissue	Mean (SAICAr in hAdsl)	Mean (SAICAr in WT)	Mean (S-Ado in hAdsl)	Mean (S-Ado in WT)	Fold change of SAICAr (hAdsl vs WT)	SAICAr_ p.value (hAdsl vs WT)	Fold change of S-Ado (hAdsl vs WT)	S-Ado_ p.value (hAdsl vs WT)	Fold change of SAICAr in hAdsl mice (females vs males)	SAICAr_ p.value (Female vs Male)	Fold change of S-Ado in hAdsl mice (females vs males)	S-Ado_ p.value (Female vs Male)
HR	Heart	0.67	0.63	0.72	0.69	1.09	0.94	1.06	0.97	0.85	0.39	0.84	0.57
KD	Kidney	0.64	0.57	0.48	0.68	1.12	0.31	0.73	0.23	1.43	0.14	0.94	0.87
LN	Lung	0.73	0.52	0.82	0.61	1.42	0.006	1.37	0.02	0.94	0.61	0.90	0.5
LV	Liver	0.97	0.49	0.76	0.81	1.96	0.000001	0.94	0.7	1.64	0.00004	1.15	0.43
SM	Skeletal muscle	0.74	0.92	0.59	0.75	0.88	0.6	0.92	0.73	1.28	0.41	2.29	0.08
SP	Spleen	0.67	0.60	0.51	0.73	1.13	0.27	0.70	0.19	1.34	0.19	1.42	0.16
CR	Cerebrum	1.66	0.82	1.59	1.49	2.06	0.00000004	1.06	0.32	1.36	0.002	1.16	0.22

Suppl. Table 3. Levels of SAICAr and S-Ado in 16 brain regions and the cerebellum of hAdsl and WT littermate mice, sexes separated.

Sex	Tissue abbr.	Tissue	Mean (SAICAr in hAdsl)	Mean (SAICAr in WT)	Mean (S-Ado in hAdsl)	Mean (S-Ado in WT)	Fold change of SAICAr (hAdsl vs WT)	SAICAr_ p.value (hAdsl vs WT)	Fold change of S-Ado (hAdsl vs WT)	S-Ado_ p.value (hAdsl vs WT)
Female	AMG	Amygdala	1.11	0.21	0.46	0.18	5.36	0.0002	2.51	0.000004
Male	AMG	Amygdala	0.66	0.22	0.34	0.20	3.01	0.008	1.68	0.04
Female	dHP	Dorsal hippocampus	0.84	0.29	0.65	0.36	2.85	0.0002	1.82	0.004
Male	dHP	Dorsal hippocampus	0.62	0.21	0.62	0.28	2.92	0.01	2.21	0.02
Female	dSTR	Dorsal straitum	1.59	0.63	0.56	0.30	2.54	0.0000002	1.85	0.002
Male	dSTR	Dorsal straitum	1.26	0.64	0.58	0.32	1.98	0.007	1.84	0.09
Female	ENT	Entorhinal cortex	0.86	0.33	0.31	0.26	2.64	0.01	1.21	0.63
Male	ENT	Entorhinal cortex	0.64	0.20	0.26	0.16	3.12	0.003	1.63	0.04
Female	FRP	Frontal pole	1.23	0.42	0.87	0.38	2.96	0.00009	2.31	0.00002
Male	FRP	Frontal pole	0.86	0.44	0.60	0.41	1.97	0.003	1.45	0.06
Female	HY	Hypothalamus	0.55	0.26	0.42	0.31	2.09	0.00008	1.34	0.07
Male	HY	Hypothalamus	0.52	0.27	0.40	0.31	1.91	0.0002	1.29	0.16
Female	MC	Motor cortex	0.57	0.30	0.34	0.26	1.92	0.000006	1.31	0.04
Male	MC	Motor cortex	0.51	0.24	0.38	0.26	2.13	0.0006	1.46	0.08
Female	mPFC	Medial prefrontal cortex	1.09	0.26	0.60	0.29	4.14	0.000002	2.09	0.000007

Male	mPFC	Medial prefrontal cortex	0.70	0.34	0.49	0.26	2.05	0.15	1.87	0.03
Female	NAc	Nucleus accumbens	0.92	0.39	0.41	0.23	2.39	0.00006	1.74	0.02
Male	NAc	Nucleus accumbens	0.73	0.36	0.34	0.19	2.04	0.003	1.78	0.01
Female	OB	Olfactory bulb	0.92	0.46	0.99	0.71	2.00	0.0001	1.39	0.02
Male	OB	Olfactory bulb	0.68	0.46	1.02	0.61	1.47	0.03	1.66	0.03
Female	SSC	Somatosencory cortex	0.71	0.37	0.43	0.42	1.92	0.004	1.02	0.94
Male	SSC	Somatosencory cortex	0.72	0.28	0.44	0.34	2.54	0.0006	1.28	0.41
Female	TH	Thalamus	0.65	0.36	0.65	0.70	1.84	0.0008	0.94	0.7
Male	TH	Thalamus	0.56	0.35	0.70	0.64	1.60	0.12	1.10	0.66
Female	VC	Visual cortex	1.50	0.28	0.84	0.28	5.43	0.00004	3.00	0.000008
Male	VC	Visual cortex	0.98	0.27	0.56	0.29	3.60	0.007	1.98	0.05
Female	vHP	Ventral hippocampus	1.60	0.36	0.92	0.38	4.51	0.0000009	2.42	0.00005
Male	vHP	Ventral hippocampus	0.95	0.35	0.69	0.43	2.69	0.02	1.61	0.09
Female	CBN	Cerebellar nuclei	0.25	0.17	0.23	0.23	1.41	0.08	1.01	0.99
Male	CBN	Cerebellar nuclei	0.16	0.16	0.15	0.19	1.04	0.91	0.79	0.42
Female	CBX	Cerebellar cortex	1.10	0.81	0.48	0.54	1.36	0.02	0.90	0.56
Male	CBX	Cerebellar cortex	0.99	0.82	0.51	0.58	1.21	0.15	0.88	0.4
Female	CB	Cerebellum	1.36	1.12	2.13	2.56	1.21	0.14	0.83	0.43

Male	CB	Cerebellum	1.34	1.08	1.95	2.26	1.24	0.08	0.86	0.24

Suppl. Table 4. Genetic correlations between 35 "distilled" phenotypic factors and succinyladenosine levels in CSF.

Factor	Phenotype	GWAS Catalog identifier	Genetic correlation	Standard error	P-value
F1	Anxiety and nervousness	GCST90309336	0.0116	0.1081	0.9149
F2	Depressive symptomatology	GCST90309337	-0.0483	0.0796	0.544
F3	Clinical anxiety and depression	GCST90309338	-0.0298	0.0893	0.739
F4	Urbanicity	GCST90309339	0.072	0.0976	0.4605
F5	Occupation and workplace environment	GCST90309340	-0.0629	0.0787	0.4242
F6	Smoking and associated risk behaviours	GCST90309341	-0.0437	0.067	0.5143
F7	BMI and adiposity	GCST90309342	-0.0055	0.049	0.9111
F9	Trauma	GCST90309343	0.0439	0.073	0.5476
F10	Education attainment	GCST90309344	0.0305	0.0552	0.5811
F11	Respiratory disease	GCST90309345	-0.0205	0.0531	0.6991
F12	Hypertension	GCST90309346	-0.0886	0.0512	0.0833
F13	Use of dietary supplements	GCST90309347	-0.0357	0.0775	0.6447
F14	Joint pain	GCST90309348	-0.1076	0.0702	0.1254
F15	Social and economic stability	GCST90309349	-0.0392	0.0778	0.6142
F16	Coronary artery disease	GCST90309350	0.0901	0.0742	0.2243
F17	Office and retail occupations	GCST90309351	-0.1938	0.1806	0.2833
F18	Chronic pain	GCST90309352	-0.0705	0.0664	0.2889
F19	Bone density	GCST90309353	0.0316	0.0583	0.5878
F20	Severe, life-threatening illness	GCST90309354	0.0033	0.0689	0.9618
F21	Body size	GCST90309355	-0.0011	0.0415	0.9793
F22	Alcohol use and misuse	GCST90309356	0.0635	0.0851	0.4557
F23	Physical activity	GCST90309357	0.0366	0.0667	0.5834
F24	Eye autorefraction and astigmatism	GCST90309358	0.0231	0.1116	0.8362
F25	Eye conditions	GCST90309359	0.0057	0.0967	0.9527
F26	Intraocular pressure	GCST90309360	0.0101	0.0703	0.8854
F27	Age-related deterioration	GCST90309361	-0.0904	0.0709	0.2025
F28	Diabetes	GCST90309362	0.0355	0.0651	0.5859
F29	Comfortable retirement	GCST90309363	-0.0125	0.0738	0.8658
F30	Inflammatory markers and poor diet	GCST90309364	-0.1049	0.0625	0.0932
F31	Gastrointestinal issues	GCST90309365	-0.0715	0.0786	0.3634
F32	Hearing	GCST90309366	-0.0805	0.1345	0.5494
F33	Living near London	GCST90309367	0.149	0.1051	0.1562
F34	Cognition and processing speed	GCST90309368	0.0162	0.075	0.8288
F35	Neutrophil and lymphocytes in blood	GCST90309369	0.008	0.0509	0.8747
F36	Handedness	GCST90309370	-0.0843	0.108	0.4349

References:

1. V. Stepanova *et al.*, Reduced purine biosynthesis in humans after their divergence from Neandertals. *Elife* **10**, e58741 (2021).
2. T. Pluskal, S. Castillo, A. Villar-Briones, M. Oresic, MZmine 2: modular framework for processing, visualizing, and analyzing mass spectrometry-based molecular profile data. *BMC Bioinformatics* **11**, 395 (2010).
3. O. D. Myers, S. J. Sumner, S. Li, S. Barnes, X. Du, One Step Forward for Reducing False Positive and False Negative Compound Identifications from Mass Spectrometry Metabolomics Data: New Algorithms for Constructing Extracted Ion Chromatograms and Detecting Chromatographic Peaks. *Anal Chem* **89**, 8696-8703 (2017).
4. A. Kiryk *et al.*, IntelliCage as a tool for measuring mouse behavior - 20 years perspective. *Behav Brain Res* **388**, 112620 (2020).
5. H. P. Lipp *et al.*, IntelliCage: the development and perspectives of a mouse- and user-friendly automated behavioral test system. *Front Behav Neurosci* **17**, 1270538 (2023).
6. Z. Fan *et al.*, Using the tube test to measure social hierarchy in mice. *Nat Protoc* **14**, 819-831 (2019).
7. R. M. Deacon, Measuring the strength of mice. *J Vis Exp* 10.3791/2610, e2610 (2013).
8. J. P. Dougherty, D. A. Springer, M. C. Gershengorn, The Treadmill Fatigue Test: A Simple, High-throughput Assay of Fatigue-like Behavior for the Mouse. *J Vis Exp* 10.3791/54052, e54052 (2016).
9. E. Hoffman, S. J. Winder, A Modified Wire Hanging Apparatus for Small Animal Muscle Function Testing. *PLoS Curr* **8**, ecurrents.md.1e2bec4e78697b78697b78690ff78680ea78625a78691d78638be (2016).
10. M. Imamura *et al.*, MondoA deficiency enhances sprint performance in mice. *Biochem J* **464**, 35-48 (2014).
11. G. Owendoff *et al.*, Optimization and construct validity of approaches to preclinical grip strength testing. *J Cachexia Sarcopeni* **14**, 2439-2445 (2023).
12. B. Zhang *et al.*, Gut Microbiota Dysbiosis Induced by Decreasing Endogenous Melatonin Mediates the Pathogenesis of Alzheimer's Disease and Obesity. *Front Immunol* **13,** 900132 (2022).
13. M. C. De Rosa *et al.*, Gene expression atlas of energy balance brain regions. *JCI Insight* **6,** e149137 (2021).
14. Z. Koenig *et al.*, A harmonized public resource of deeply sequenced diverse human genomes. *Genome Res* **34**, 796-809 (2024).
15. D. E. Cook, E. C. Andersen, VCF-kit: assorted utilities for the variant call format. *Bioinformatics* **33**, 1581-1582 (2017).
16. M. D. Rasmussen, M. J. Hubisz, I. Gronau, A. Siepel, Genome-wide inference of ancestral recombination graphs. *PLoS Genet* **10**, e1004342 (2014).
17. A. H. Vaughn, R. Nielsen, Fast and Accurate Estimation of Selection Coefficients and Allele Histories from Ancient and Modern DNA. *Mol Biol Evol* **41**, msae156 (2024).
18. T. Jespersen, M. Grunnet, K. Angelo, D. A. Klærke, S. P. Olesen, Dual-function vector for protein expression in both mammalian cells and oocytes. *Biotechniques* **32**, 536-538 (2002).
19. R. Ågren, K. Sahlholm, Voltage-Dependent Dopamine Potency at D-Like Dopamine Receptors. *Frontiers in Pharmacology* **11**, 581151 (2020).
